# A novel mode of WRKY1 regulating PR1-mediated immune balance to defend against powdery mildew in apple

**DOI:** 10.1186/s43897-024-00141-z

**Published:** 2025-03-05

**Authors:** Liming Lan, Lifang Cao, Lulu Zhang, Weihong Fu, Changguo Luo, Chao Wu, Xianqi Zeng, Shenchun Qu, Xinyi Yu, Wenyi Deng, Xu Xu, Binhua Cai, Sanhong Wang

**Affiliations:** 1 College of Horticulture, Nanjing Agricultural University, Nanjing, 210095 China; 2 Institute of Pomology, Guizhou Academy of Agricultural Sciences, Guiyang, 550006 China

**Keywords:** Apple powdery mildew, Salicylic acid, PR1, Plant immunity, Immune balance, WRKY40-NPR3g module, NPR1, TGA2c variants, Alternative splicing, BTB-POZ domain, EPS1, WRKY1, Immune defense mechanism

## Abstract

Powdery mildew (PM), caused by the biotrophic fungus *Podospharea leucotricha*, poses a significant threat to apple production. Salicylic acid (SA) signaling plays a crucial role in enhancing resistance to biotrophic pathogens. While PR1, a defense protein induced by SA, is essential for plant immunity, its excessive accumulation can be detrimental. However, the mechanism of PR1-mediated immune balance remains unclear. This study identified a key transcription factor, WRKY1, which enhances the SA accumulation by modulating the SA biosynthesis gene *EPS1*, while simultaneously regulating the WRKY40-NPR3g module to prevent sustained *PR1* expression caused by continuous SA accumulation. Specifically, the transcription factor WRKY40 upregulates *NPR3g* expression, and NPR3g interacts with NPR1 in an SA-dependent manner. Then, two TGA2c variants that interact with NPR1 to activate *PR1* expression were identified: canonical TGA2c-1 and alternative splicing of TGA2c-2 with an exon deletion. SA does not influence the NPR1-TGA2c-1 interaction but is essential for the NPR1-TGA2c-2 interaction. Notably, NPR3g reduces PR1 levels by selectively disrupting the NPR1-TGA2c-2 complex through competition for the BTB-POZ domain of NPR1. In conclusion, this study identifies a novel mechanism by which WRKY1 modulates PR1-mediated immune balance to defend against PM.

**Supplementary Information:**

The online version contains supplementary material available at 10.1186/s43897-024-00141-z.

## Core

In apple, a key transcription factor, WRKY1, regulating immune balance was identified, which promotes the SA biosynthesis while preventing excessive PR1 expression induced by continuous SA accumulation.

## Gene & Accession Numbers

Sequence information of apple genes in this study are from the GDDH13 v1.1 reference genome in the GDR database (https://www.rosaceae.org/species/malus/malus_x_domestica/genome_GDDH13_v1.1), and the accession numbers can be found in Supplementary Table S1. Arabidopsis genes involved in this study are from the TAIR database (https://www.arabidopsis.org/), and the accession numbers can be searched in the database using the gene names.

## Introduction

Powdery mildew (PM), a widespread disease in apple cultivation, is caused by the biotrophic fungus *Podospharea leucotricha* (Gañán et al. 2020). This pathogen predominantly affects buds, leaves, new shoots, and young fruits, significantly impacting tree health and diminishing both fruit yield and quality (Zhang et al. 2021; Papp et al. 2016).

Salicylic acid (SA) plays a crucial role in the plant immune system, essential for defense against biotrophic pathogens (An et al., 2011). In *Arabidopsis thaliana*, two primary pathways contribute to SA biosynthesis: the phenylalanine ammonia lyase (PAL) and the isochorismate synthase (ICS) pathways. The PAL pathway accounts for approximately 10% of pathogen-induced SA, while the ICS pathway serves as the predominant source, contributing around 90% (Huang et al. 2010; Wang et al. 2022). In the ICS pathway, AtICS1 converts plastidic branch acids to isochorismate (Wildermuth et al., 2001), which is then transported to the cytoplasm by the MATE transporter AtEDS5 (Serrano et al. 2013). Subsequently, aminotransferase AtPBS3 catalyzes the formation of the glutamate-9-isobranched acid conjugate. Finally, acyltransferase AtEPS1 cleaves this conjugate to produce SA (Rekhter et al. 2019; Torrens-Spence et al. 2019).

SA accumulation plays a crucial role in the upregulation of pathogenesis-related proteins, particularly AtPR1, in *Arabidopsis thaliana* (Horvath et al., 1996; Dong 2004; Uquillas, 2004; Muthamilarasan and Prasad 2013). The molecular mechanism underlying SA-mediated AtPR1 induction follows a specific pathway. Pathogen-induced SA cleaves the disulfide bonds within AtNPR1 oligomers, enabling ligand-bound AtNPR1 monomers to translocate into the nucleus. Once there, these monomers interact with AtTGA2 to regulate *AtPR1* gene transcription (Kesarwani et al. 2007). Additionally, Cul3, functioning as an E3 ubiquitin ligase, facilitates the proteasomal degradation of target proteins. In conditions of elevated SA, AtNPR3, a homolog of AtNPR1, acts as an adaptor to promote the interaction between AtCul3 and AtNPR1, thus targeting AtNPR1 for ubiquitin-dependent degradation (Backer et al. 2019; Fu et al. 2012).

WRKY transcription factors play a crucial role in plant responses to biotic and abiotic stresses (Pieterse et al. 2012). Initially identified in 1994 (Ishiguro et al., 1994), these factors are characterized by a conserved domain of approximately 60 amino acids (Mahiwal et al. 2023). They regulate the transcription of various target genes by binding to specific DNA sequences (Yang et al., 2001). Numerous WRKYs regulate defense against pathogens by participating in the SA signaling pathway in *Arabidopsis* (Xu et al. 2006; Li et al. 2004). For example, AtWRKY28 enhances SA biosynthesis by acting as a positive regulator of the *AtICS1* gene (Wang et al. 2015). In contrast, AtWRKY70 and AtWRKY54 co-suppress SA biosynthesis, thereby contributing to stress response (Li et al. 2013). Yu et al. (2001) demonstrated that SA-induced WRKY-binding proteins specifically recognize the W-box element in the *AtNPR1* promoter, thus activating the expression of downstream pathogenesis-related genes. Furthermore, AtWRKY38 and AtWRKY62 function as negative regulators of basal plant defenses by repressing *PR1* gene expression (Kim et al. 2008).

Pathogen-induced SA activates the accumulation of PR1 protein, which is central to plant defense responses, yet this activation often coincides with the inhibition of normal plant growth and development (Thomma et al. 2001; Dong 2004). However, research on the mechanisms regulating immune balance by controlling PR1 levels remains limited. In this study, we observed that *PR1* expression levels did not continue to increase with SA accumulation following PM attack, suggesting a regulatory mechanism that maintains immune balance by controlling PR1 levels. To elucidate this mechanism, we initially analyzed the expression profiles of six WRKYs post-PM infection from previous research, identifying WRKY40 as a promising candidate. Subsequently, we found that WRKY40 is involved in regulating *PR1* expression. DNA affinity purification sequencing (DAP-seq) analysis of WRKY40 revealed numerous potential downstream genes. Further experimentation confirmed that WRKY40 positively regulates *NPR3g* expression. The molecular mechanism by which SA-regulated NPR3g suppresses *PR1* expression was then delineated. Additionally, the transcription factor WRKY1 was found to positively regulate the WRKY40-NPR3g module. Finally, the molecular mechanism by which WRKY1 promoted SA biosynthesis was elucidated. In conclusion, our study uncovered a novel mechanism by which WRKY1 regulates SA-mediated immune balance to defend against PM attack.

## Results

### Revealing a mechanism regulating SA-mediated immune balance by controlling PR1 levels following PM infection

**Fig. 1:**
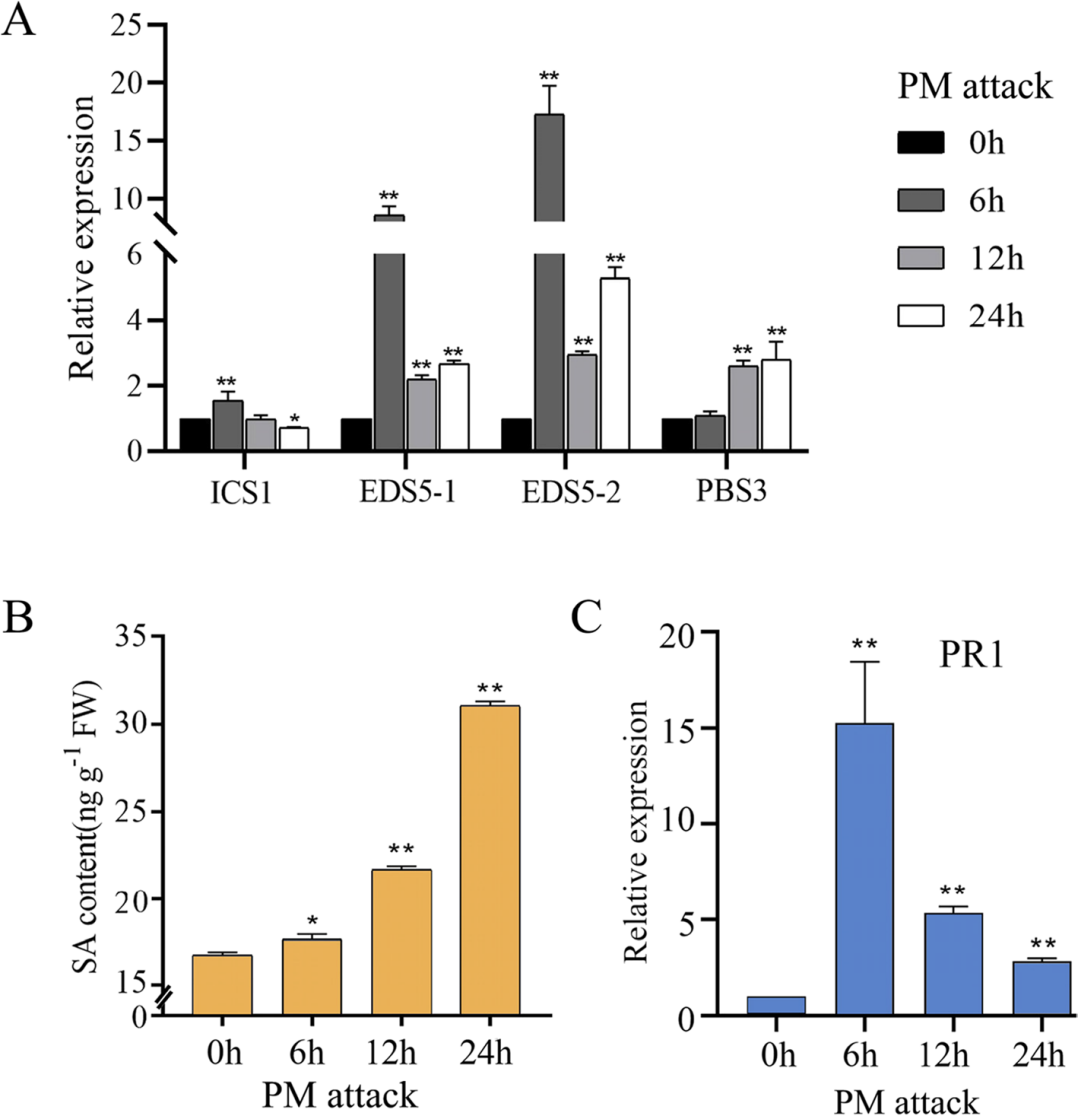
There be a mechanism to regulate SA-mediated immune balance by controlling PR1 levels after PM attack. **A** RT-qPCR analysis of the expression of SA biosynthesis genes following PM attack. **B** Changes in endogenous SA content in plants after PM attack. **C** RT-qPCR analysis of the expression of the *PR1* gene following PM attack. The data represent the means and standard deviations of three independent replicate experiments. Asterisks (*) indicate significant differences from the control (Student’s t test, **P < 0.01)

As illustrated in Supplemental Fig. 1, we inoculated PM pathogens into tissue culture seedlings to enhance reproduction and preservation. The SA signaling pathway is widely recognized for its role in mediating resistance to biotrophic pathogens, with PR1 serving as a primary SA-induced defense protein. To elucidate the mechanism by which the SA signaling pathway regulates PM resistance, we examined the expression of SA biosynthetic genes, endogenous SA levels, and *PR1* gene expression following PM inoculation. Our results demonstrated significant upregulation of SA synthesis genes *ICS1*, *EDS5-1*, *EDS5-2*, and *PBS3* in response to PM infection (Fig. 1A). Quantitative analysis of endogenous SA levels revealed that within 24 h post-inoculation with PM, SA concentrations exhibited a gradual increase over time (Fig. 1B). In contrast, *PR1* expression peaked at 6 h and subsequently declined, returning to basal levels by 24 h (Fig. 1C). This discrepancy between *PR1* expression and SA accumulation suggests the presence of a regulatory mechanism that modulates PR1 levels to maintain SA-mediated immune homeostasis.

### Transcription factor WRKY40, a negative regulator of PM resistance, is involved in regulating the expression of the *PR1 *gene

**Fig. 2:**
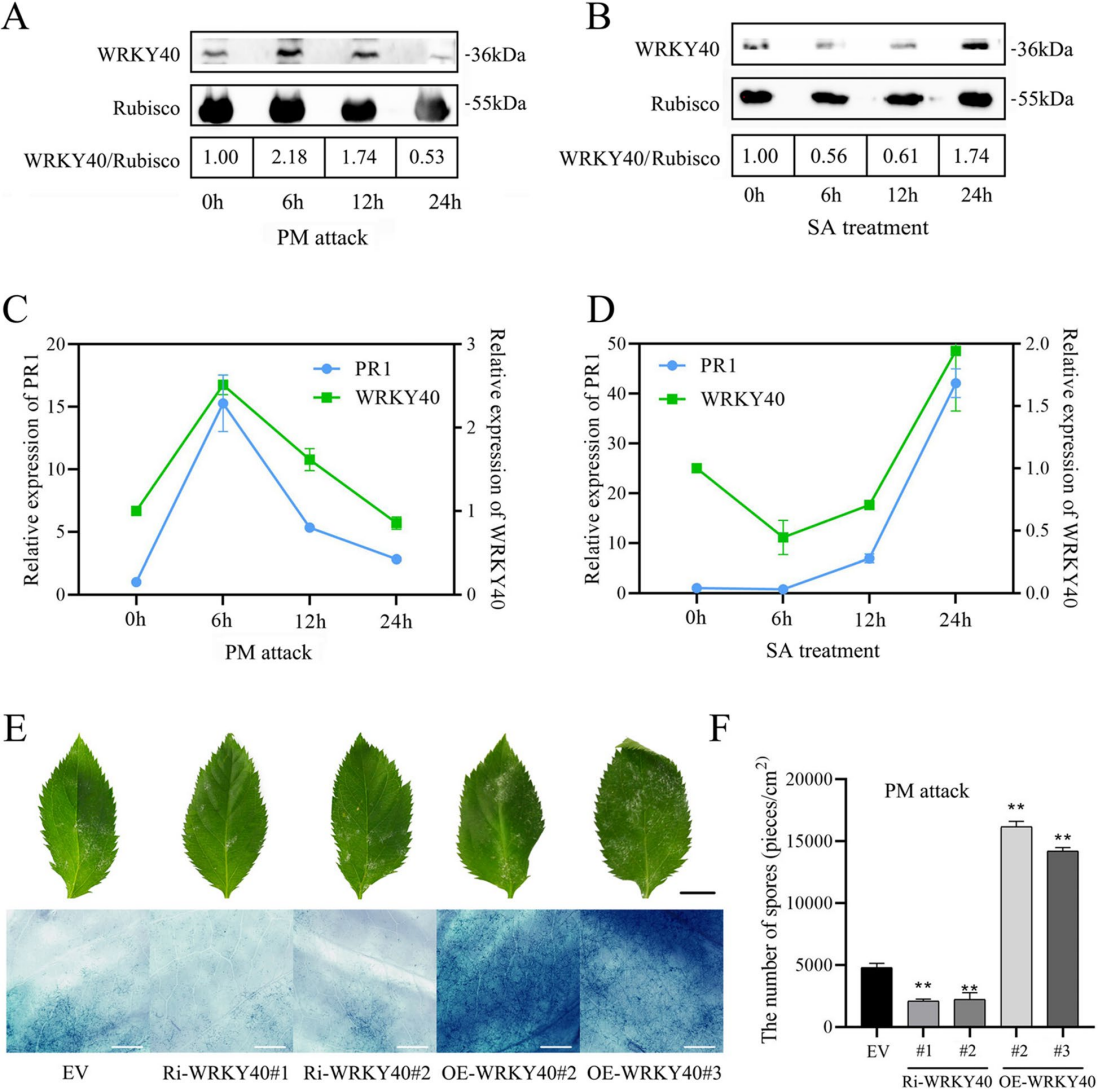
Transcription factor WRKY40, a negative regulator of PM resistance, is involved in regulating the expression of the *PR1* gene. **A** and **B** Western Blotting analysis of WRKY40 protein accumulation following PM attack (**A**) and SA treatment (**B**). Rubisco was used as Loading control. WRKY40/Rubisco is the ratio of gray scale values of protein bands. **C** and **D** RT-qPCR analysis of the expression patterns of *WRKY40* and* PR1* genes after PM attack (**C**) and SA treatment (**D**). **E** Observations of the overall powdery mildew lesions on leaf surfaces were made 7 days after PM inoculation. Subsequently, leaves were stained with trypan blue and examined for spores and mycelia using a super-depth stereomicroscope. Ri-*WRKY40* represents RNAi-silenced *WRKY40* plants, OE-*WRKY40* represents *WRKY40*-overexpressing plants, and EV represents empty vector control plants. Black and white scale bars represent 1 cm and 1 mm, respectively. **F** Spore counts and leaf area measurements were taken for all leaves of the entire plant 7 days after PM inoculation to calculate spore density per unit leaf area. The data represent the means and standard deviations of three independent replicate experiments. Asterisks (*) indicate significant differences from the control (Student’s t test, **P < 0.01)

In our previous investigation, we identified six WRKY transcription factors responsive to PM infection: WRKY40, WRKY2, WRKY3, WRKY22, WRKY26, and WRKY70 (Lan et al. 2021). Quantitative RT-PCR analysis demonstrated significant upregulation of these genes following PM challenge, with the *WRKY40* gene exhibiting the most pronounced increase (Supplemental Fig. 2). Western blotting confirmed a corresponding elevation in WRKY40 protein levels post-infection, mirroring the transcriptional upregulation (Fig. 2A). Consequently, this study focuses on WRKY40 to elucidate its regulatory mechanism.

To elucidate WRKY40's function in SA-mediated defense responses, we examined WRKY40 protein levels following SA treatment, observing notable variations in its accumulation (Fig. 2B). Furthermore, our results demonstrated a correlated expression pattern between *WRKY40* and *PR1* in response to both PM challenge and SA treatment (Fig. 2C, D). Together, these findings indicate a potential regulatory role for WRKY40 in *PR1* gene expression.

To assess WRKY40's regulatory function in PM resistance, we generated transgenic plants with RNAi-mediated silencing (Ri-*WRKY40*) or overexpression (OE-*WRKY40*). These plants were validated using GUS staining and RT-qPCR (Supplemental Figs. 3 and 4). Seven days after PM inoculation, Ri-*WRKY40* plants exhibited significantly lower pathogen loads on leaf surfaces compared to control plants, while OE-*WRKY40* plants showed increased pathogen loads. Trypan blue staining and quantitative spore counts per unit leaf area confirmed this pattern (Fig. 2E, F). Furthermore, *WRKY40* was found to be nuclear-localized (Supplemental Fig. 5). These results collectively indicate that the transcription factor WRKY40 functions as a negative regulator of PM resistance.

### Genome-wide identification of WRKY40 binding sites and target genes by DAP-seq

**Fig. 3:**
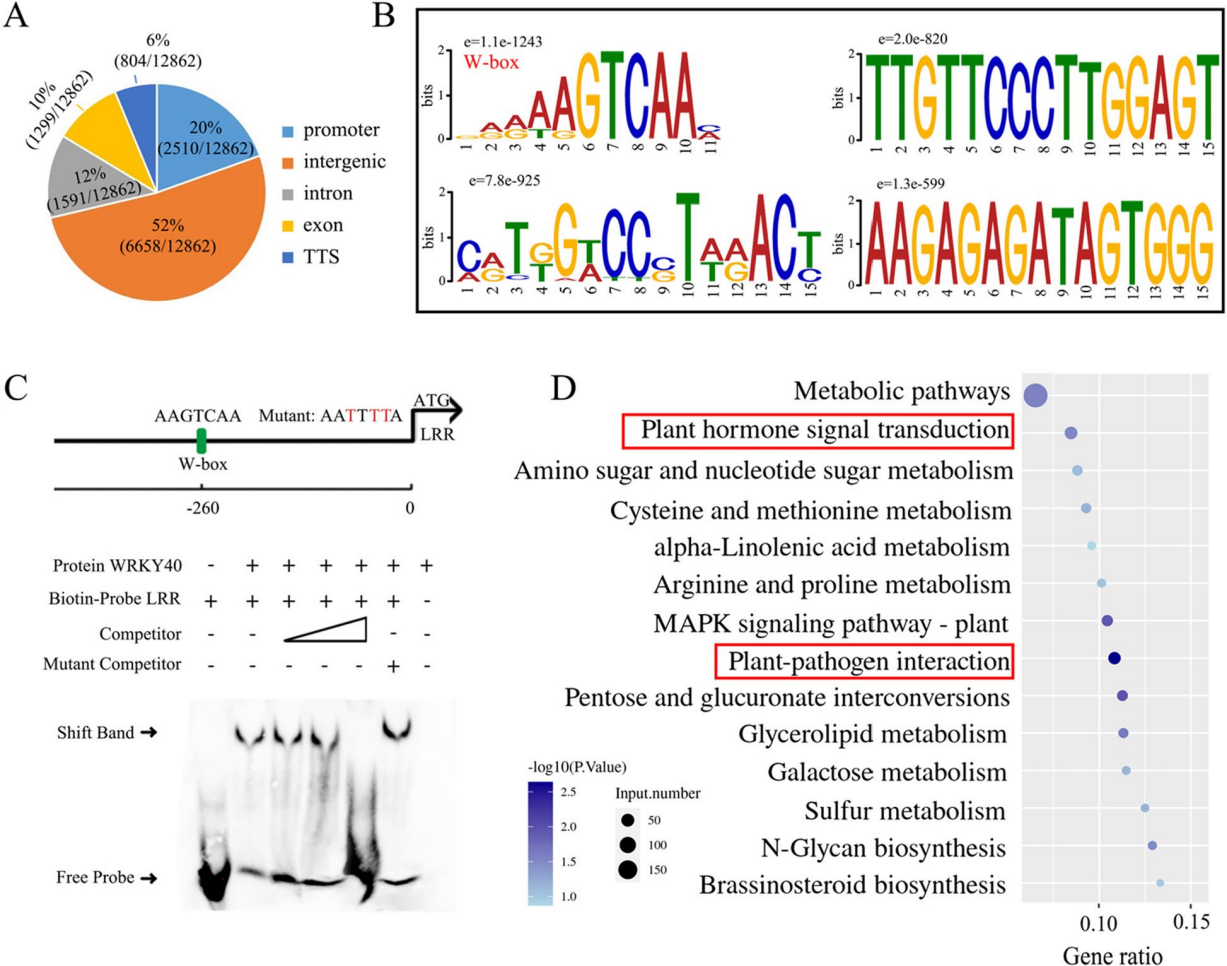
Genome-wide identification of WRKY40 binding sites and target genes by DAP-seq. **A** The number of bound peaks by WRKY40 obtained by DAP-seq and their distribution in the whole genome. **B** The identified binding motifs of WRKY40 protein by MEME-ChIP. The core sequence of “AAGTCAA” was substantially enriched among the WRKY40 binding regions (e-value = 1.1e-1243) and was named W-box. **C** The position of the W-box element in the promoter and its mutated sequence are displayed. EMSA showed WRKY40 binds to the W-box element of LRRpro (MD17G1204800). Competitor refers to the unlabeled biotin probe. The mutated W-box element sequence corresponds to that in Figure **C**. **D** The top KEGG-enriched terms of WRKY40-bound genes by DAP-seq. The two pathways highlighted in red are of our interest

To elucidate the direct transcriptional targets of WRKY40, DAP-seq was employed to reveal its genome-wide DNA binding profiles (Bartlett et al. 2017). Analysis demonstrated that 72% of *WRKY40* binding peaks were located in promoter or intergenic regions, with 12% in introns, 10% in exons, and 6% in the transcription termination site (TTS) regions (Fig. 3A). Utilizing the MEME suite, a significantly enriched W-box motif ("AAGTCAA") was identified within the *WRKY40* binding sites (e value = 1.1e-1243; Fig. 3B). Among the four candidate motifs identified, the W-box exhibited the most significant e value, leading to its selection as the primary criterion for subsequent identification of WRKY40 downstream targets.

Electrophoretic mobility shift assays (EMSA) utilizing His-tagged WRKY40 protein and W-box DNA probes confirmed WRKY40's specific binding to the W-box motif (Fig. 3C). This specificity was demonstrated by reduced binding in the presence of cold competitor DNA and sustained binding with mutant competitors. Additionally, Kyoto Encyclopedia of Genes and Genomes (KEGG) pathway analysis of the predicted WRKY40 targets revealed an enrichment in hormone signaling and plant-pathogen interaction pathways (Fig. 3D). In summary, these findings suggest that WRKY40 selectively interacts with the W-box element to regulate genes involved in hormone-mediated plant-pathogen responses.

### NPR3g, a positively regulated downstream target of WRKY40, negatively regulates PM resistance by repressing *PR1* expression

To investigate WRKY40's involvement in *PR1* regulation, we focused on downstream proteins known to influence *PR1* expression. Considering that AtNPR3/4 are established repressors of *PR1* (Wang et al. 2020), we utilized AtNPR3/4 as a template for homology alignment in the apple genome. This analysis yielded seven *NPR3/4-like* genes, which were subsequently designated as *NPR3a-g*, followed by phylogenetic tree analyses (Supplemental Fig. 6).

**Fig. 4:**
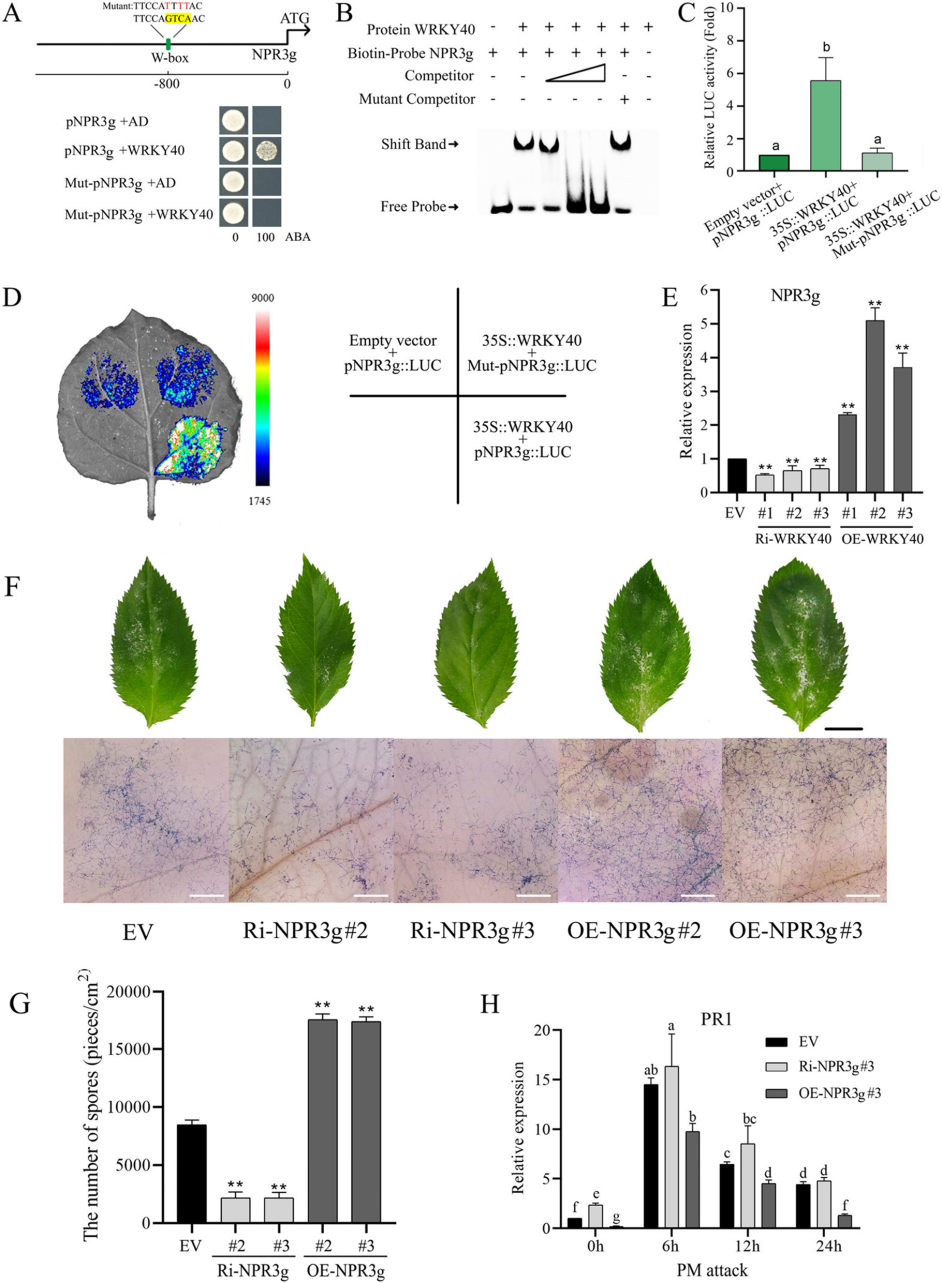
NPR3g, a direct downstream gene of WRKY40, negatively regulates PM resistance by repressing PR1 expression. **A** The position of the W-box element in the promoter and its mutated sequence are displayed. Y1H analysis indicates that WRKY40 specifically binds to the W-box element of the *NPR3g* promoter. Selection medium contains SD/-Leu medium supplemented with 0 and 100 ng/mL aureobasidin A. **B** EMSA analysis demonstrates the specific binding of WRKY40 to the W-box element of the *NPR3g* promoter. Competitor refers to the unlabeled biotin probe. The mutated W-box element sequence corresponds to that in Figure **A**. **C** and **D** LUC analysis indicates that WRKY40 positively regulates the activity of the *NPR3g* promoter-driven LUC. The mutated W-box element sequence corresponds to that in Figure **A**. **E** Expression levels of *NPR3g* in *WRKY40* transgenic plants. Ri-*WRKY40 *represents RNAi-silenced *WRKY40* plants, OE-*WRKY40* represents *WRKY40*-overexpressing plants, and EV represents empty vector control plants. **F** Observations of the overall powdery mildew lesions on leaf surfaces were made 7 days after PM inoculation. Subsequently, leaves were stained with trypan blue and examined for spores and mycelia using a super-depth stereomicroscope. Ri-*NPR3g* represents RNAi-silenced *NPR3g* plants, OE-*NPR3g* represents *NPR3g*-overexpressing plants, and EV represents empty vector control plants. Black and white scale bars represent 1 cm and 1 mm, respectively. **G** Spore counts and leaf area measurements were taken for all leaves of the entire plant 7 days after PM inoculation to calculate spore density per unit leaf area. **H** RT-qPCR indicates that the expression of *PR1* in *NPR3g* transgenic lines compared to EV after PM attack. The data represent the means and standard deviations of three independent replicate experiments. Asterisks (*) indicate significant differences from the control (Student’s t test, **P < 0.01)

Analysis of the DAP-seq database for W-box elements in the promoter regions of *NPR3a-g* genes confirmed *NPR3g* as a direct transcriptional target of WRKY40 through a series of experimental validations. Figure 4A illustrates the identification of a W-box element at -800 bp upstream of the *NPR3g* initiation codon. Yeast one-hybrid (Y1H) assays demonstrated that WRKY40 bound specifically to the W-box of the *NPR3g* promoter, evidenced by growth on selective medium, which was abrogated with a mutated W-box (Fig. 4A). EMSA revealed that WRKY40 specifically bound the biotin probe of the *NPR3g* promoter containing the W-box element. The addition of the cold competition probe significantly inhibited the binding signal, while the mutated competition probe had no effect on the binding signal (Fig. 4B). Luciferase (LUC) assays showed that WRKY40 significantly enhanced *NPR3g* promoter activity, an effect negated by W-box mutations (Fig. 4C, D). Furthermore, *NPR3g* was significantly suppressed in Ri-*WRKY40* plants and significantly up-regulated in OE-*WRKY40* plants compared to control plants (Fig. 4E). These findings collectively demonstrate that WRKY40 specifically binds to the W-box element of *NPR3g* and positively regulates its expression.

Finally, we examined the influence of NPR3g on PM resistance. Transgenic plants with RNAi-mediated silencing (Ri-*NPR3g*) or overexpression (OE-*NPR3g*) were validated using GUS staining and RT-qPCR (Supplemental Figs. 7 and 8). Seven days post-PM inoculation, Ri-*NPR3g* plants exhibited significantly reduced pathogen loads on leaf surfaces compared to control plants, while OE-*NPR3g* plants showed increased pathogen loads. This pattern was corroborated by trypan blue staining and quantitative spore counts per unit leaf area (Fig. 4F, G). Additionally, we analyzed the expression of *PR1* in Ri-*NPR3g* and OE-*NPR3g* plants. At various time points following PM attack, *PR1* was significantly up-regulated in Ri-*NPR3g* plants and down-regulated in OE-*NPR3g* plants compared to control plants (Fig. 4H). Collectively, these findings indicate that NPR3g negatively regulates PM resistance by suppressing *PR1* expression.

### NPR3g, a protein that consists of a BTB-POZ domain only, interacts with NPR1 in an SA-regulated manner to regulate NPR1 activity

**Fig. 5:**
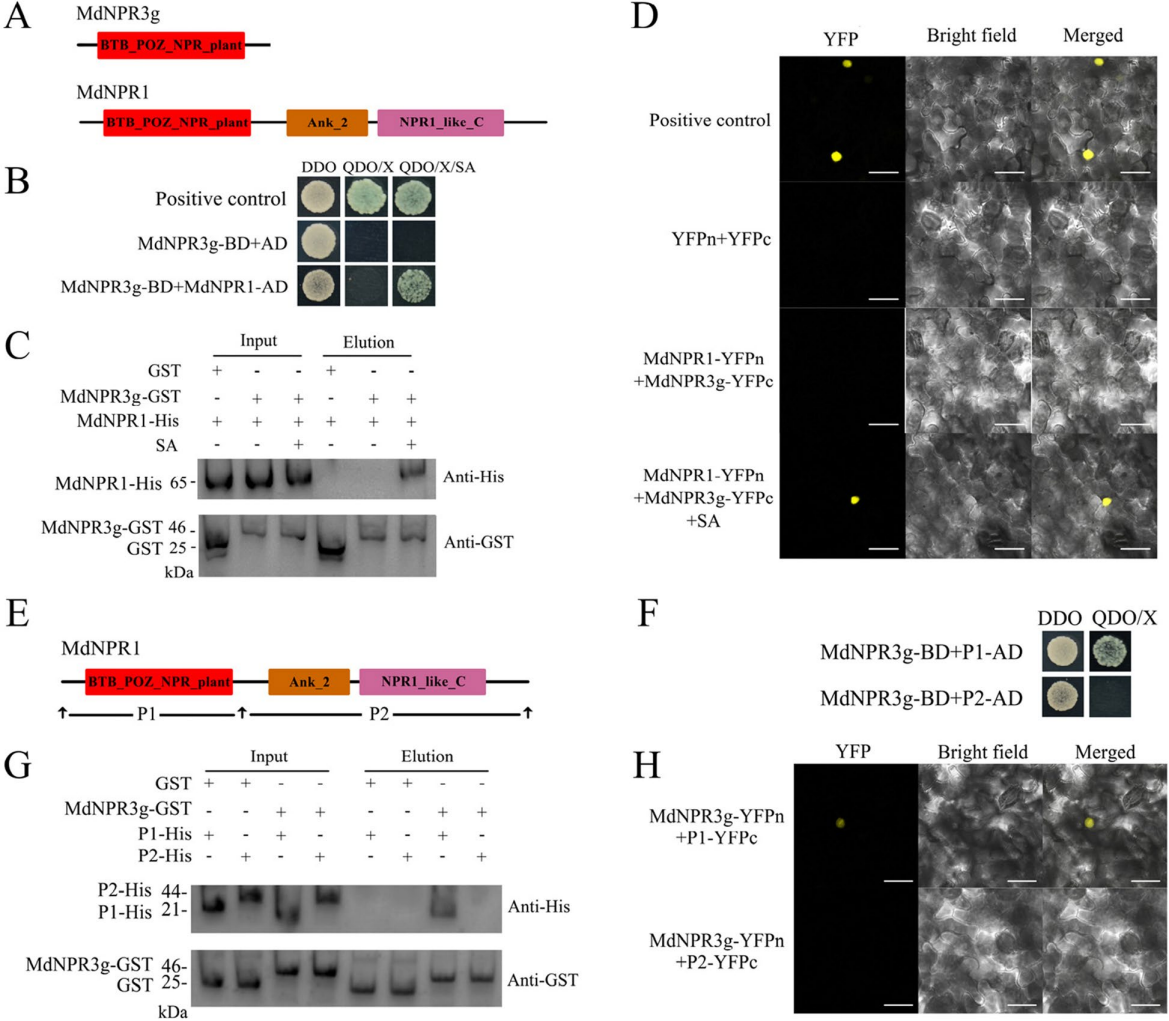
The interaction between NPR3g and NPR1 is SA-dependent, with NPR3g selectively binding to the BTB-POZ domain of NPR1. **A** Schematic representation of the domain structures of NPR3g and NPR1. NPR3g consists solely of a BTB-POZ domain, while NPR1 comprises BTB-POZ, Ank2, and NPR1-like domains. **B** Yeast two-hybrid (Y2H) analysis demonstrates that NPR1 interacts with NPR3g in an SA-regulated manner. Positive controls are pGBKT7-53 and pGADT7-T. DDO refers to SD/-Leu-Trp medium; QDO/X denotes SD/-Leu-Trp-His-Ade medium supplemented with X-α-gal; QDO/X/SA is QDO/X medium with added SA. **C** GST-pulldown shows an SA-regulated interaction between NPR1 and NPR3g. NPR3g carries a GST tag, and NPR1 carries a His tag. **D** Bimolecular fluorescence complementation (BiFC) indicates an SA-regulated interaction between NPR1 and NPR3g. Positive controls are the combinations of MdMYB308L-YFPn and MdbHLH33-YFPc. Scale bar is 50 μm. **E** The NPR1 protein is truncated into two parts, P1 and P2. P1 includes the N-terminal BTB-POZ domain, and P2 includes the C-terminal Ank2 and NPR1-like domains. **F**, **G**, **H** Y2H (**F**), GST-pulldown (**G**), and BiFC (**H**) results indicate that NPR3g selectively binds to P1. The experimental design corresponds to figures **B**, **C**, and **D**, with the difference being the absence of SA

Given that the WRKY40-NPR3g module suppresses PM resistance by repressing *PR1* expression, we aimed to elucidate the mechanism of NPR3g. Typically, AtNPR3/4 modulates *PR1* expression by influencing AtNPR1 activity (Wang et al. 2020). Consequently, we investigated the molecular interactions between NPR3g and NPR1. Structural analysis revealed that NPR3g possesses a BTB/POZ domain, while NPR1 comprises BTB/POZ, Ank2, and NPR1-like domains (Fig. 5A). Yeast two-hybrid assays (Y2H) demonstrated that the presence of SA mitigated the growth inhibition of strains co-expressing NPR3g-BD and NPR1-AD on QDO medium (Fig. 5B). Additionally, in vitro GST-pulldown assays with recombinant proteins confirmed a direct SA-dependent interaction between NPR3g and NPR1 (Fig. 5C). Bimolecular fluorescence complementation (BiFC) assay showed that co-expression of NPR3g-YFPc and NPR1-YFPn in *N. benthamiana* leaves under SA treatment resulted in nuclear YFP fluorescence, which was absent in leaves transformed with individual constructs (Fig. 5D). Collectively, these findings indicate an SA-regulated interaction between NPR3g and NPR1.

To identify the NPR1 domain involved in the interaction with NPR3g, we divided NPR1 into N-terminal P1 (BTB/POZ domain) and C-terminal P2 (Ank2 + NPR1-like domain) segments (Fig. 5E). Subsequent yeast two-hybrid (Y2H), GST-pulldown, and BiFC assays demonstrated that NPR3g specifically interacts with the BTB/POZ domain of NPR1 (Fig. 5F, G, H). These findings indicate that the BTB-POZ domain of NPR1 is crucial for the NPR1-NPR3g interaction, suggesting that SA binding triggers a conformational change in NPR1, enabling the BTB-POZ domain-mediated interaction with NPR3g.

### TGA2c-2, an exon deletion transcript of TGA2c, interacts with NPR1 in an SA-regulated manner to promote PR1 expression

**Fig. 6:**
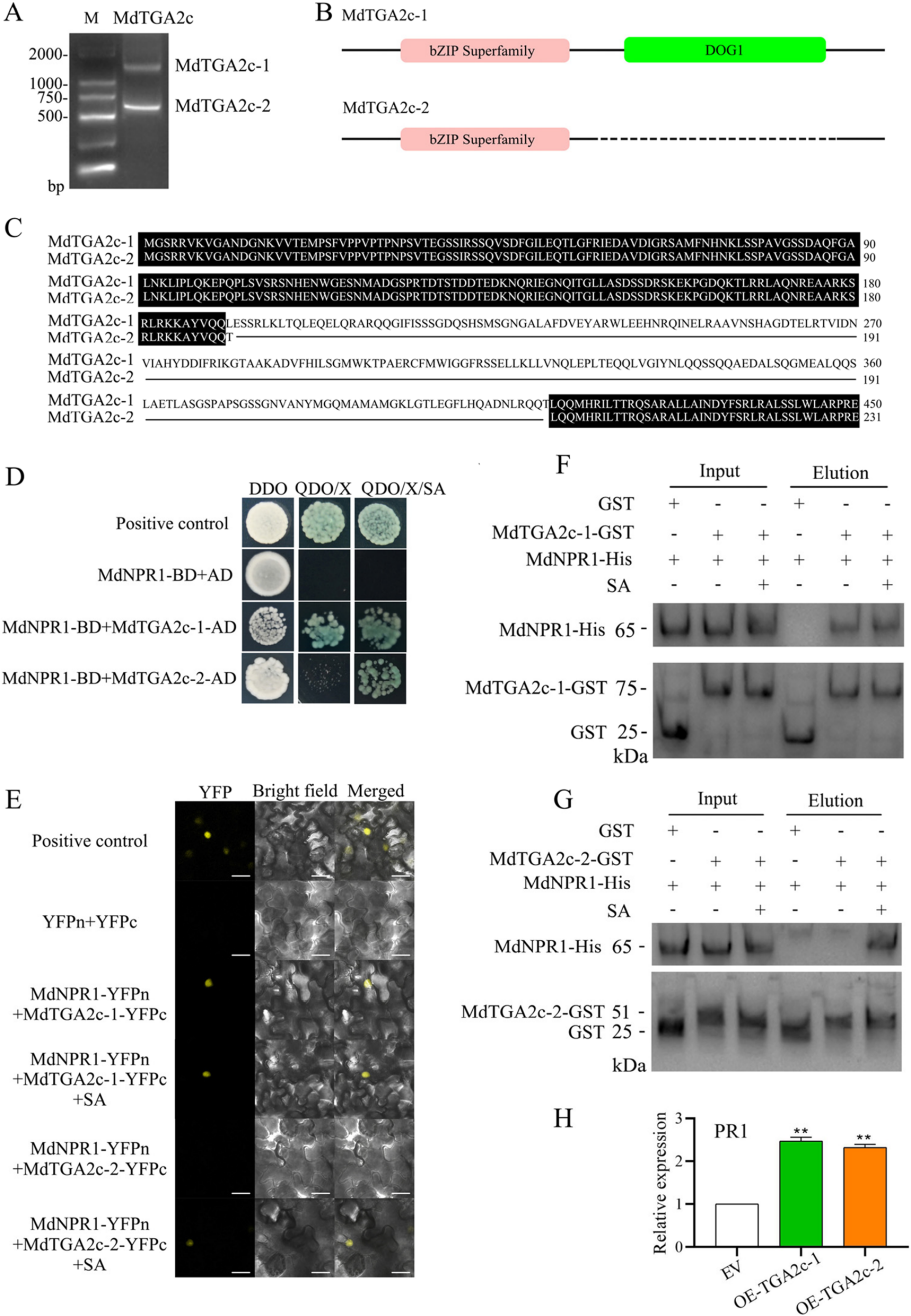
Two different transcripts of TGA2c, TGA2c-1 and TGA2c-2, positively regulate *PR1* expression by interacting with NPR1 in an SA-independent and dependent manner, respectively. **A** Agarose gel electrophoresis illustrates the CDS sequence lengths of the two transcripts of* TGA2c*, *TGA2c-1 *and* TGA2c-2*. M represents a 2000 bp ladder. **B** Schematic representation of the domain structures of TGA2c-1 and TGA2c-2 proteins. TGA2c-1 consists of BZIP and DOG1 domains, while TGA2c-2 contains only a BZIP domain. **C** BLAST results for the protein sequences of TGA2c-1 and TGA2c-2. **D** Yeast two-hybrid (Y2H) results indicate that the interaction between NPR1 and TGA2c-1 is SA-independent, whereas the interaction between NPR1 and TGA2c-2 is SA-dependent. Positive controls are pGBKT7-53 and pGADT7-T. DDO refers to SD/-Leu-Trp medium; QDO/X denotes SD/-Leu-Trp-His-Ade medium supplemented with X-α-gal; QDO/X/SA is QDO/X medium with added SA. **E** Bimolecular fluorescence complementation (BiFC) results show that the interaction between NPR1 and TGA2c-1 is SA-independent, and the interaction between NPR1 and TGA2c-2 is SA-dependent. Positive controls are the combinations of MdMYB308L-YFPn and MdbHLH33-YFPc. Scale bar is 50 μm. **F** and **G** GST-pulldown results demonstrate that the interaction between NPR1 and TGA2c-1 is SA-independent (**F**), and the interaction between NPR1 and TGA2c-2 is SA-dependent (**G**). Both TGA2c-1/2 carry GST tags, while NPR1 carries a His tag. **H** RT-qPCR indicates that the expression of *PR1* is significantly upregulated in overexpression lines of TGA2C-1/2 compared to EV. OE-TGA2c-1/2 denotes overexpression plants of TGA2C-1/2, and EV represents empty vector control plants. The data represent the means and standard deviations of three independent replicate experiments. Asterisks (*) indicate significant differences from the control (Student’s t test, **P < 0.01)

Given that AtNPR1 and AtTGA2/5/6 form a complex to activate *PR1* expression (Kesarwani et al. 2007), we hypothesized that NPR3g, which interacts with NPR1 in an SA-regulated manner, might interfere with the binding of other proteins, such as TGA2/5/6, to NPR1. To investigate this, we searched the apple genome for TGA2/5/6 homologs using AtTGA2/5/6 as a reference. This search identified six *TGA2/5/6-like* genes, which we designated *TGA2a-f*, and conducted phylogenetic analysis (Supplemental Fig. 9). Additionally, our analysis revealed two distinct transcripts of TGA2c: the canonical TGA2c-1 and an alternatively spliced variant, TGA2c-2, with an exon deletion. TGA2c-1 contains both BZIP and DOG1 domains, while TGA2c-2 contains only the BZIP domain (Fig. 6A-C).

Y2H assays revealed that strains containing NPR1-BD and TGA2c-1-AD exhibited normal growth on QDO medium regardless of SA presence, while strains with NPR1-BD and TGA2c-2-AD required SA for normal growth (Fig. 6D). In vitro GST-pulldown assays confirmed direct interactions between NPR1 and both TGA2c-1 (SA-independent) and TGA2c-2 (SA-dependent) (Fig. 6F, G). Nuclear YFP fluorescence was observed in *N. benthamiana* leaves upon co-expression of TGA2c-1-YFPc and NPR1-YFPn, irrespective of SA treatment. However, TGA2c-2-YFPc and NPR1-YFPn co-expression resulted in fluorescence only under SA treatment (Fig. 6E). These results indicate that SA does not affect the NPR1-TGA2c-1 interaction but is crucial for the NPR1-TGA2c-2 interaction. Furthermore, considering the conformational change in NPR1 upon SA binding (Fig. 5), the BTB-POZ domain of NPR1 appears to be essential for the NPR1-TGA2c-2 interaction.

To elucidate the role of TGA2c-1/2 in *PR1* gene regulation, we generated overexpressing plants for both genes and analyzed them using GUS staining and RT-qPCR (Supplemental Fig. 10, 11). RT-qPCR results revealed a significant upregulation of *PR1* in OE-*TGA2c-1* and OE-*TGA2c-2* plants compared to control plants (Fig. 6H). These findings indicate that both TGA2c-1 and TGA2c-2 positively modulate *PR1* gene expression.

### NPR3g suppresses *PR1* expression by disrupting the NPR1-TGA2c-2 protein interaction in an SA-dependent manner

**Fig. 7:**
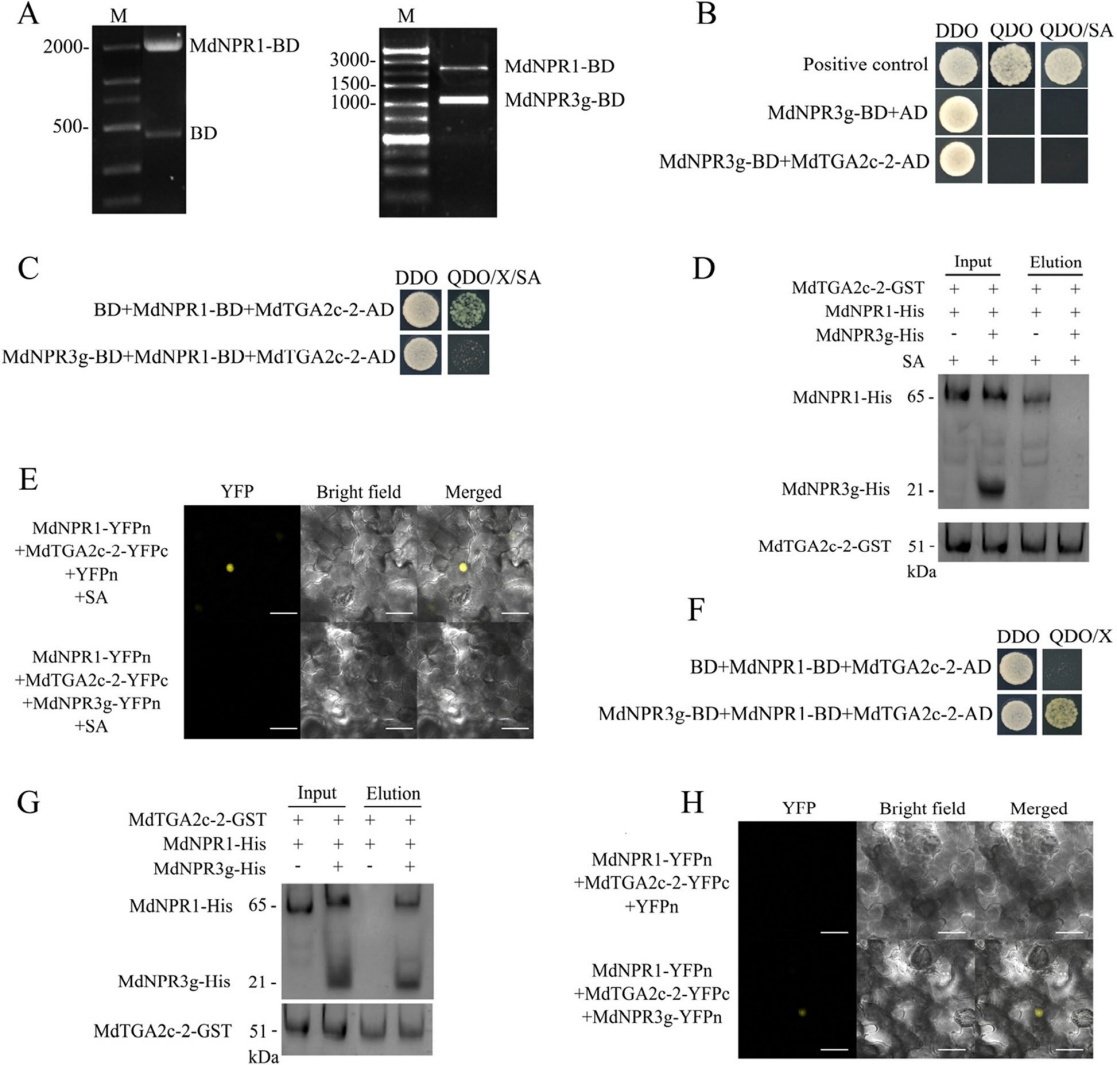
NPR3g disrupts the NPR1-TGA2c-2 protein interaction in an SA-dependent manner, and NPR3g facilitates the interaction between NPR1-TGA2c-2 in the absence of SA. **A** Agarose gel electrophoresis reveals PCR outcomes from yeast lysates co-transformed with NPR1-BD and BD, and NPR1-BD and NPR3g-BD. **B** Yeast two-hybrid (Y2H) assays indicate no interaction between NPR3g and TGA2c-2. DDO refers to SD/-Leu-Trp medium; QDO/X denotes SD/-Leu-Trp-His-Ade medium supplemented with X-α-gal; QDO/X/SA is the same medium with additional SA. Positive controls are pGBKT7-53 and pGADT7-T. **C** Yeast three-hybrid (Y3H) results show NPR3g inhibits the SA-dependent interaction of NPR1-TGA2c-2. The pGBKT7 vector was used to express NPR3g as a third protein, with empty pGBKT7 as a control. **D** GST-pulldown results demonstrate NPR3g's inhibition of the SA-dependent interaction between NPR1-TGA2c-2. TGA2c-2 carries a GST tag, while NPR1 and NPR3g both bear His tags. **E** Bimolecular fluorescence complementation (BiFC) confirms NPR3g's inhibition of the SA-dependent interaction between NPR1-TGA2c-2. TGA2c-2 is fused to YFPc, and NPR1 and NPR3g are fused to YFPn. Scale bar represents 50 μm. **F**, **G**, **H** Y3H (**F**), GST-pulldown (**G**), and BiFC (**H**) results indicate that NPR3g facilitates the interaction between NPR1-TGA2c-2 in the absence of SA, corresponding to experiments in **C**, **D**, and **E**, except without SA supplementation

To evaluate the impact of NPR3g on the NPR1-TGA2c-2 interaction in an SA-dependent manner, we implemented a yeast three-hybrid (Y3H) assay utilizing NPR3g-BD, NPR1-BD, and TGA2c-2-AD. Co-transformation of NPR3g-BD and NPR1-BD was confirmed through colony PCR (Fig. 7A). Initially, Y2H assays demonstrated no direct interaction between NPR3g and TGA2c-2 (Fig. 7B), suggesting that NPR3g influences the NPR1-TGA2c-2 interaction exclusively through NPR1.

Y3H assays revealed that strains containing NPR3g-BD, NPR1-BD, and TGA2c-2-AD exhibited growth inhibition on QDO medium under SA conditions (Fig. 7C). In vitro GST-pulldown assays utilizing recombinant proteins confirmed that NPR3g inhibits the NPR1-TGA2c-2 interaction under SA conditions (Fig. 7D). No YFP fluorescence was detected upon co-expression of NPR3g-YFPn, NPR1-YFPn, and TGA2c-2-YFPc in *N. benthamiana* leaves following SA treatment (Fig. 7E). These results suggest that NPR3g inhibits the NPR1-TGA2c-2 interaction in an SA-dependent manner. Considering the critical role of NPR1's BTB-POZ domain in interactions with both NPR3g and TGA2c-2 (Figs. 5 and 6), we hypothesize that NPR3g exerts its inhibitory effect by competing with TGA2c-2 for binding to NPR1's BTB-POZ domain.

Notably, yeast strains co-expressing NPR3g with NPR1 and TGA2c-2 exhibited normal growth on QDO medium without SA (Fig. 7F). This observation was further supported by GST-pulldown and BiFC assays (Fig. 7G, H). These findings indicate that NPR3g, which contains a BTB/POZ domain, can enhance the NPR1-TGA2c-2 interaction in the absence of SA. These results corroborate the previously established conclusion that the BTB-POZ domain is essential for the NPR1-TGA2c-2 interaction; SA binding to NPR1 induces conformational changes, enabling its BTB-POZ domain to interact with other proteins.

### Transcription factor WRKY1 functions to repress PR1 levels by positively modulating the WRKY40-NPR3g module

In conclusion, this study elucidates the SA-dependent mechanism of *PR1* repression by the WRKY40-NPR3g module, prompting further investigation into the upstream regulatory elements of this module.

**Fig. 8:**
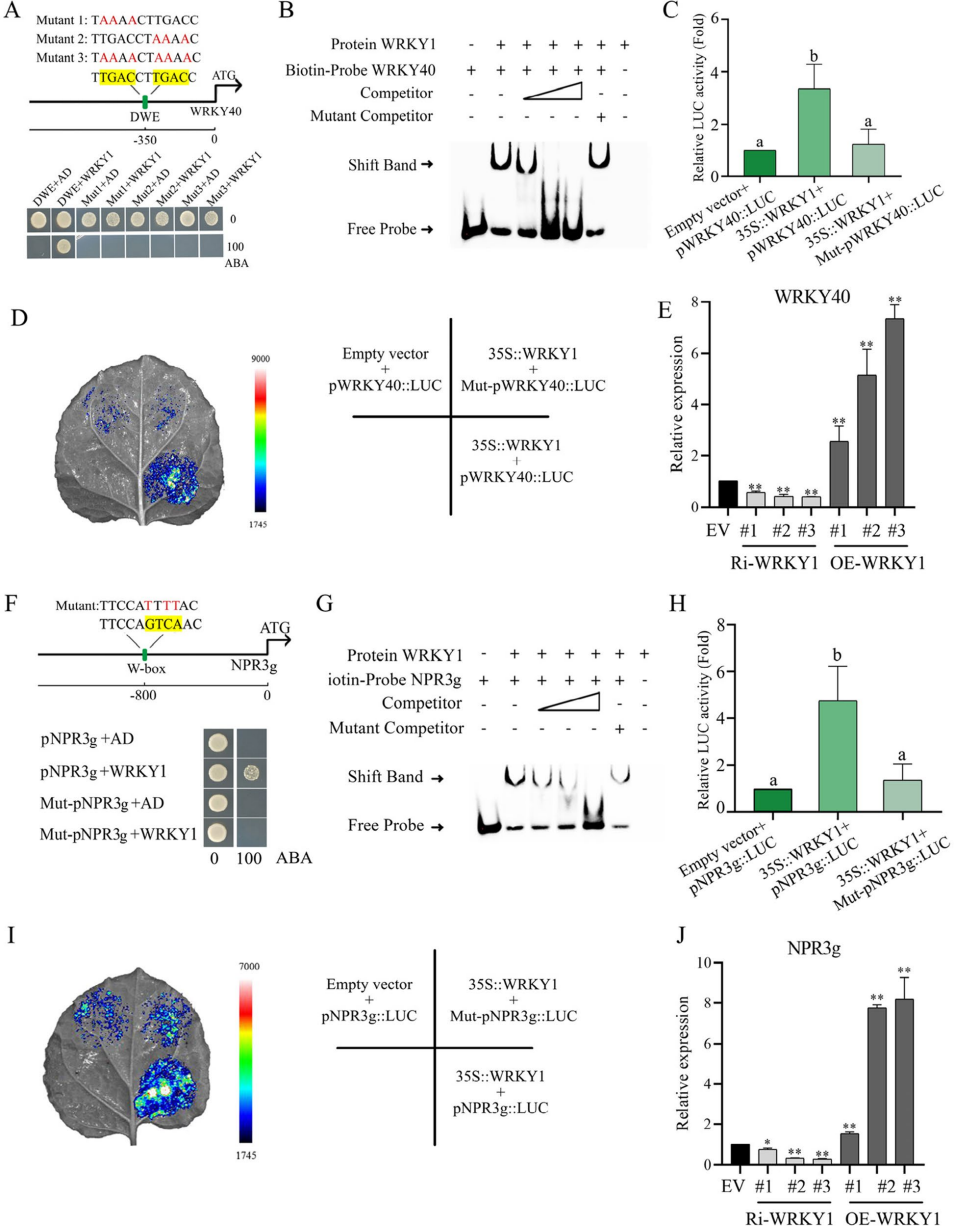
WRKY1 positively regulates *WRKY40* and *NPR3g* expression. **A** The position of the DWE element in the promoter and its mutated sequence are displayed. Y1H analysis indicates that WRKY1 specifically binds to the DWE element of the *WRKY40* promoter. Selection medium contains SD/-Leu medium supplemented with 0 and 100 ng/mL aureobasidin A. **B** EMSA analysis demonstrates the specific binding of WRKY1 to the DWE element of the *WRKY40* promoter. Competitor refers to the unlabeled biotin probe. The mutated DWE element sequence corresponds to Mutant 3 in Figure **A**. **C** and **D** LUC analysis indicates that WRKY1 positively regulates the activity of the *WRKY40* promoter-driven LUC. The mutated DWE element sequence corresponds to Mutant 3 in Figure **A**. **E** Expression levels of *WRKY40* in *WRKY1* transgenic plants. Ri-*WRKY1* represents RNAi-silenced *WRKY1* plants, OE-*WRKY1* represents *WRKY1*-overexpressing plants, and EV represents empty vector control plants. **F** The position of the W-box element in the promoter and its mutated sequence are displayed. Y1H analysis indicates that WRKY1 specifically binds to the W-box element of the *NPR3g* promoter. Selection medium contains SD/-Leu medium supplemented with 0 and 100 ng/mL aureobasidin A. **G** EMSA analysis demonstrates the specific binding of WRKY1 to the W-box element of the *NPR3g* promoter. Competitor refers to the unlabeled biotin probe. The mutated W-box element sequence corresponds to that in Figure **F**. **H** and **I** LUC analysis indicates that WRKY1 positively regulates the activity of the *NPR3g* promoter-driven LUC. The mutated W-box element sequence corresponds to that in Figure **F**. **J** Expression levels of *NPR3g* in *WRKY1* transgenic plants. Ri-*WRKY1* represents RNAi-silenced *WRKY1* plants, OE-*WRKY1* represents *WRKY1*-overexpressing plants, and EV represents empty vector control plants. The data represent the means and standard deviations of three independent replicate experiments. Asterisks (*) indicate significant differences from the control (Student’s t test, **P < 0.01)

To elucidate the upstream regulatory mechanisms, we conducted an analysis of the *WRKY40* promoter and identified a DWE element located 350 bp upstream of the initiation codon. This element comprises two consecutive W-box elements (Fig. 8A). Considering that WRKY subfamily I genes bind to DWE elements in pepper (Liu et al. 2021), we postulated the existence of analogous WRKY subfamily I genes in apple.

Subsequently, we identified WRKY1 as an upstream regulator of WRKY40. Y1H assays demonstrated that yeast containing WRKY1-AD and DWE-pAbAi grew normally on selective medium, while growth was inhibited with a mutated DWE element (Fig. 8A). EMSA revealed that WRKY1 specifically bound the biotin probe of the *WRKY40* promoter containing the DWE element. The addition of the cold competition probe significantly inhibited the binding signal, whereas the addition of the mutated competition probe had no effect on the binding signal (Fig. 8B). LUC assays showed that WRKY1 significantly upregulated *WRKY40* promoter activity, and DWE element mutations reduced this activity (Fig. 8C, D). Transgenic plants with RNAi silencing or overexpression of *WRKY1*, confirmed by GUS staining and RT-qPCR (Supplemental Fig. 12, 13), exhibited downregulation of *WRKY40* expression in silenced lines and upregulation in overexpressing lines compared to controls (Fig. 8E). These findings indicate that WRKY1 specifically binds to the DWE element of *WRKY40* and positively regulates its expression.

Y1H and EMSA assays unexpectedly demonstrated WRKY1's specific binding to the W-box element of the *NPR3g* promoter (Fig. 8F, G). Additionally, LUC assays and RT-qPCR confirmed that WRKY1 positively regulates *NPR3g* expression (Fig. 8H-J). These findings indicate that WRKY1 specifically binds to the W-box element of *NPR3g* and enhances its expression. Consequently, WRKY1 functions to suppress PR1 levels in an SA-dependent manner by positively modulating the WRKY40-NPR3g module.

### WRKY1 enhances SA biosynthesis by positively regulating the *EPS1* gene to defend against PM

**Fig. 9:**
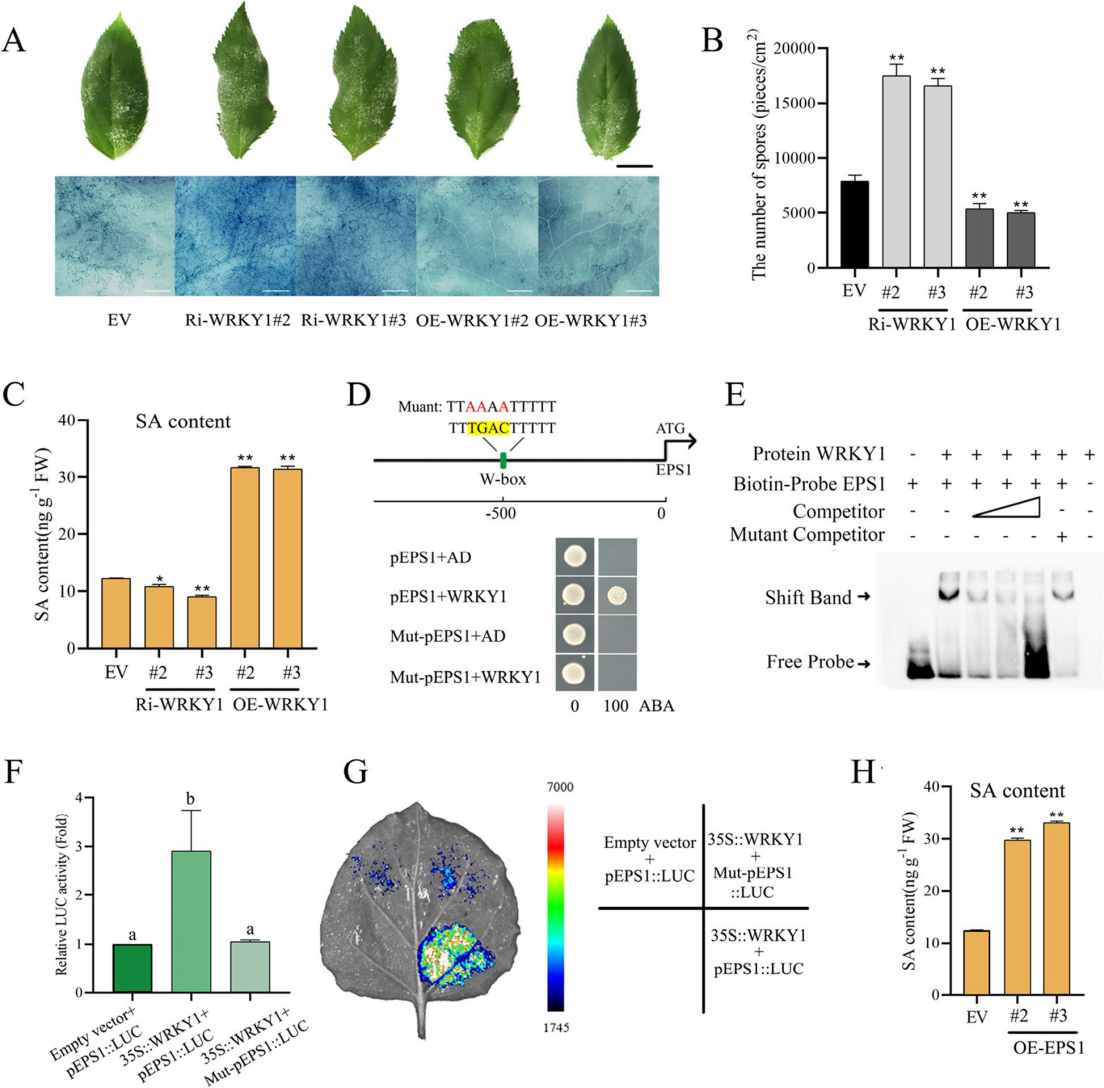
WRKY1 can also promote SA biosynthesis by positively regulating the *EPS1* gene to defend against PM. **A** Observations of the overall powdery mildew lesions on leaf surfaces were made 7 days after PM inoculation. Subsequently, leaves were stained with trypan blue and examined for spores and mycelia using a super-depth stereomicroscope. Ri-*WRKY1* represents RNAi-silenced *WRKY1* plants, OE-*WRKY1* represents *WRKY1*-overexpressing plants, and EV represents empty vector control plants. Black and white scale bars represent 1 cm and 1 mm, respectively. **B** Spore counts and leaf area measurements were taken for all leaves of the entire plant 7 days after PM inoculation to calculate spore density per unit leaf area. **C** Endogenous SA content in *WRKY1*-silenced and overexpressing lines. **D** The position of the W-box element in the promoter and its mutated sequence are displayed. Y1H analysis indicates that WRKY1 specifically binds to the W-box element of the *EPS1* promoter. Selection medium contains SD/-Leu medium supplemented with 0 and 100 ng/mL aureobasidin A. **E** EMSA analysis demonstrates the specific binding of WRKY1 to the W-box element of the *EPS1* promoter. Competitor refers to the unlabeled biotin probe. The mutated W-box element sequence corresponds to that in Figure **D**. **F** and **G** LUC analysis indicates that WRKY1 positively regulates the activity of the* EPS1* promoter-driven LUC. The mutated W-box element sequence corresponds to that in Figure **D**. **H** Endogenous SA content in *EPS1*-overexpressing lines. OE-*EPS1* represents *EPS1*-overexpressing plants, and EV represents empty vector control plants. The data represent the means and standard deviations of three independent replicate experiments. Asterisks (*) indicate significant differences from the control (Student’s t test, **P < 0.01)

This study examined the function of WRKY1 in modulating resistance to PM. Notably, 7 days post-PM inoculation, Ri-*WRKY1* plants exhibited significantly elevated pathogen loads on leaf surfaces compared to control specimens, while OE-*WRKY1* plants demonstrated reduced pathogen loads. These observations were corroborated by trypan blue staining and quantitative spore counts per unit leaf area (Fig. 9A, B). Subcellular localization analyses revealed that WRKY1 is predominantly localized in the nucleus (Supplemental Fig. 14). In conclusion, the transcription factor WRKY1 plays a positive role in regulating PM resistance.

These findings indicate that WRKY1 not only regulates the WRKY40-NPR3g module but also influences the overall immune response against PM. To further investigate this, we measured endogenous SA levels in Ri-*WRKY1* and OE-*WRKY1* plants. The results revealed significantly reduced SA levels in Ri-*WRKY1* plants and increased SA levels in OE-*WRKY1* plants compared to control plants (Fig. 9C). This result suggests that WRKY1 can enhance endogenous SA content.

To investigate the mechanism by which WRKY1 regulates SA biosynthesis, this study focused on known related genes. The *AtEPS1* gene has been previously reported to be involved in SA biosynthesis (Rekhter et al. 2019; Torrens-Spence et al. 2019). Consequently, AtEPS1 was utilized as a template for homology alignment in the apple genome, resulting in the identification of 36 *EPS1-like* genes (Supplemental Fig. 15). Subsequently, genes containing W-box elements in their promoters were selected as candidate targets for WRKY1 downstream gene validation.

Following Y1H point-to-point validation, the *EPS1* gene (MD17G1056000) was identified as a direct downstream target of WRKY1*.* As illustrated in Fig. 9D, a W-box element located 500 bp upstream of the initiation codon was examined. Strains harboring WRKY1-AD and p*EPS1*-pAbAi exhibited normal growth on selective medium, while growth was inhibited with a mutated W-box element. EMSA assays demonstrated that WRKY1 specifically binds to the biotin probe of the *EPS1* promoter containing the W-box; the addition of a cold competition probe significantly inhibited the binding signal, whereas the addition of a mutated competition probe had no effect on the binding signal (Fig. 9E). LUC assays revealed that WRKY1 significantly upregulated *EPS1* promoter-driven LUC activity, and mutation in the W-box reduced this activity (Fig. 9F, G). Moreover, overexpression of *EPS1*, confirmed by GUS staining and RT-qPCR (Supplemental Fig. 16, 17), resulted in elevated endogenous SA levels compared to control plants (Fig. 9H), indicating that EPS1 can promote SA biosynthesis. In conclusion, WRKY1 positively regulates the SA biosynthesis gene *EPS1* by binding to the W-box of its promoter.

## Discussion

**Fig. 10:**
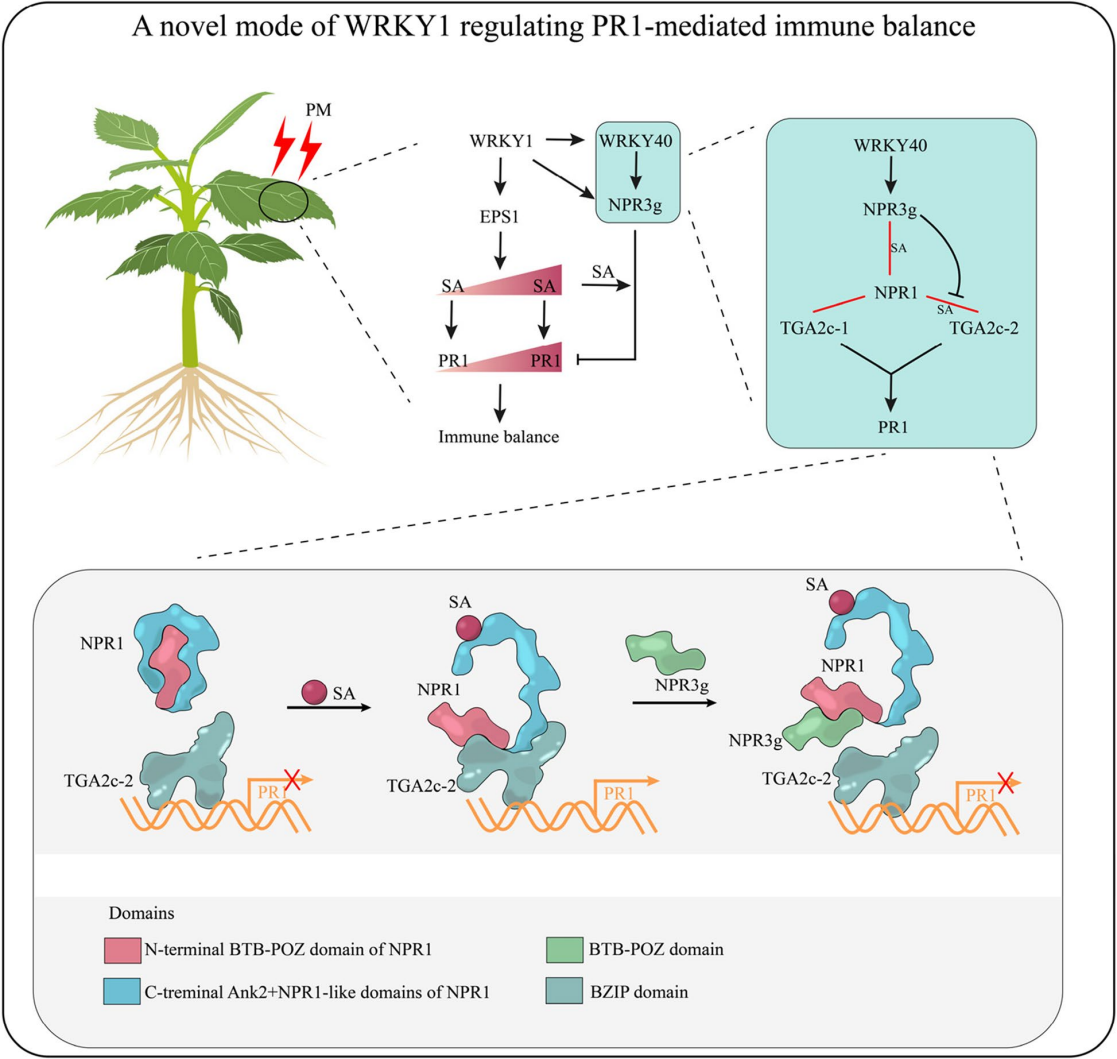
Model of WRKY1 regulating PR1-mediated immune balance in defense against powdery mildew (PM). Upon PM attack, plants activate salicylic acid (SA)-mediated defense mechanisms. WRKY1 promotes SA biosynthesis by positively regulating the SA biosynthetic gene *EPS1*, which in turn initiates PR1 protein production. As PM infection progresses, SA accumulates, leading to excessive PR1 protein accumulation, which can be detrimental to plant growth and development. To balance defense and growth, WRKY1 initiates the WRKY40-NPR3g module to suppress *PR1* expression in an SA-dependent manner. WRKY40 positively regulates *NPR3g* expression. NPR3g interacts with NPR1 in an SA-dependent manner. Two TGA2c transcripts, TGA2c-1 and TGA2c-2, both interact with NPR1 to activate *PR1* expression, with the distinction that the interaction between TGA2c-2 and NPR1 is SA-dependent. NPR3g inhibits *PR1 *expression by disrupting the TGA2c-2-NPR1 interaction. Mechanistically, the BTB-POZ domain of NPR1 is essential for its binding to both NPR3g and TGA2c-2. In other words, NPR3g competes with TGA2c-2 for binding to the BTB-POZ domain of NPR1. The red line represents protein-protein interaction

PR1 is a crucial defense protein induced by SA, though excessive accumulation of PR1 can be detrimental to plant (Thomma et al. 2001; Dong 2004). This study reveals that *PR1* expression initially increases following PM infection in conjunction with SA accumulation but subsequently declines with further SA accumulation, indicating a regulatory mechanism for immune balance. The research has identified a regulatory model involving WRKY1, which plays a significant role in this process. Specifically, WRKY1 enhances SA accumulation by modulating *EPS1*, while simultaneously regulating the WRKY40-NPR3g module to prevent sustained *PR1* expression (Fig. 10).

### The WRKY40-NPR3g module suppresses *PR1* expression by disrupting the NPR1-TGA2c-2 protein interaction in an SA-dependent manner

In *Arabidopsis thaliana*, AtNPR1 functions as a crucial receptor for SA in plant immune responses. Typically existing as an oligomer in the cytoplasm, AtNPR1 monomerizes upon SA induction and translocates to the nucleus. There, it interacts with AtTGA transcription factors to form an enhancer complex that activates the *AtPR1* gene (Kesarwani et al. 2007; Ali et al. 2018). The AtTGA subfamily, characterized by BZIP and DOG1 domains, includes AtTGA2, AtTGA5, and AtTGA6, which are involved in the regulation of SA-mediated *AtPR1* expression (Zhang et al. 2003). Although AtNPR3 is a paralog of AtNPR1, its precise role in SA signaling remains unclear. Both AtNPR1 and AtNPR3 proteins contain BTB/POZ, Ank2, and NPR1-like domains (Kumar et al. 2022). In this study, we identified a paralog of *AtNPR3* in apple, *NPR3g*, which comprises only one BTB-POZ domain and is positively regulated by WRKY40 (Figs. 4 and 5).

The BTB-POZ domain plays a crucial role in the functionality of AtNPR1 in *Arabidopsis*. This domain is described as a core structural component of the AtNPR1 protein, contributing to its various functions. SA binding induces a conformational change in AtNPR1, resulting in the release of the C-terminal transcription activation domain from the N-terminal BTB/POZ domain. This structural alteration may influence AtNPR1's interaction with other transcription factors, thereby modulating the expression of downstream genes (Kumar et al. 2022; Wu et al. 2012; Boatwright et al., 2013). In this study, we observed an intriguing SA-regulated interaction between NPR3g and NPR1. Additionally, our findings revealed that NPR3g selectively binds to the BTB-POZ domain of NPR1 (Fig. 5). Concurrently, we identified that TGA2c-2 also interacts with NPR1 in an SA-regulated manner, with the BTB-POZ domain being essential for this interaction (Fig. 6). Based on these observations, we propose that SA binding induces a conformational change in NPR1, enabling its BTB-POZ domain to interact with either NPR3g or TGA2c-2.

In *Arabidopsis*, AtNPR3 modulates AtNPR1 protein stability under high SA conditions by functioning as an adaptor for Cullin3 ubiquitin E3 ligase, which downregulates immunity (Fu et al. 2012; Backer et al. 2019; Wang et al. 2020). This study has uncovered a novel mechanism through which NPR3g suppresses PR1-mediated immunity. TGA2c-1 and TGA2c-2 are two distinct transcripts of the TGA2c transcription factor that initiate *PR1* expression. NPR3g inhibits *PR1* expression by specifically disrupting the SA-dependent interaction between TGA2c-2 and NPR1, without affecting the SA-independent interaction between TGA2c-1 and NPR1 (Fig. 7). Taken together, these findings elucidate a novel regulatory mechanism by which *NPR3g* suppresses the prolonged expression of *PR1*.

### WRKY1 regulates SA-induced *PR1* expression to mediate immune balance

SA is a crucial defense hormone, yet it can simultaneously inhibit plant growth and development (Shields et al. 2022). Plants must maintain a delicate balance between defense mechanisms and growth to combat pathogens without sacrificing developmental vigor. Consequently, the equilibrium between SA-mediated defense and growth is essential during pathogen invasion. Extensive research has elucidated the defense-growth balance mechanism, emphasizing the interaction between SA and auxin. It is well-established that the SA-auxin relationship is vital for regulating plant root apical meristem (RAM) homeostasis and growth (Navarro et al. 2006; Wang et al. 2007; Pasternak et al. 2019). SA inhibits auxin biosynthesis and signaling, reducing cell division rates in the RAM and thereby limiting root growth. Moreover, H_2_O_2_ suppresses levels of the auxin precursor tryptophan by sulfenylating TSB1, while SA inhibits CATALASE2 (CAT2) activity to increase H_2_O_2_ levels in plants upon pathogen infection (Yuan et al. 2017).

Pathogen-induced SA activates the accumulation of PR proteins, which are crucial for plant defense responses. However, this activation often conflicts with normal plant growth and development (Thomma et al. 2001; Dong 2004). Despite this, research on the mechanisms regulating immune balance by controlling PR1 accumulation levels remains limited. Our study revealed a novel phenomenon: following PM attack, *PR1* expression levels did not continue to increase with SA accumulation, suggesting an apple-specific mechanism that limits continuous *PR1* expression to maintain immune balance (Fig. 1). We identified the regulatory protein WRKY1 as a key factor underlying this mechanism. WRKY1 enhances SA accumulation by modulating the SA biosynthesis gene *EPS1* while simultaneously preventing excessive *PR1* expression through the WRKY40-NPR3g module (Figs. 8 and 9). Collectively, our findings elucidate a new regulatory model centered on WRKY1, offering novel insights into PR1-mediated immune balance and providing a foundation for the development of disease-resistant and robust plant varieties.

## Materials and methods

### PM attack and SA treatment

The cultivation conditions for apple tissue culture seedlings include a temperature of 25 °C with a photoperiod of 16 h light and 8 h darkness. Robust seedlings, grown for 30 days, were selected for PM inoculation and SA treatment. The PM inoculation method involved using forceps to hold leaves bearing conidia and rubbing them repeatedly onto the surface of the tissue culture seedlings until no white lesions were visible on the conidia-carrying leaves. The SA treatment consisted of uniformly spraying the tissue culture seedling leaves with a 0.4 mM SA solution. Following PM inoculation and SA treatment, samples were collected from entire plants at 0, 6, 12, and 24 h for RT-qPCR analysis. Seven days post-inoculation, spores from all leaves of in vitro seedlings were washed off with sterile water, and spore counts were determined using a hemocytometer. Simultaneously, photographs of these leaves were taken, and the total leaf area was measured using Photoshop, enabling the calculation of spore density per unit leaf area. Each sampling time point underwent three independent biological replicates.

### Western Blotting

Western Blotting was utilized to evaluate WRKY40 protein accumulation following PM infection and SA treatment. Robust 30-day-old seedlings were selected for PM inoculation and SA treatment. Samples were collected at 0, 6, 12, and 24 h post-treatment and rapidly frozen in liquid nitrogen. Subsequently, total protein was extracted from the samples for analysis. The WRKY40 antibody was prepared by Zoonbio Biotechnology (Song et al. 2021). Rubisco antibody served as a control. The WRKY40 antibody was applied at a 1:200 dilution, while Rubisco antibody was used at a 1:2000 dilution. Image J software was employed to quantify the grayscale values of the protein bands (Carvajal-Vergara et al. 2010).

### Determination of endogenous SA

Nanjing Ruiyuan Biotechnology Co., Ltd. quantified SA using the LC–MS/MS platform described by Izumi et al. (2009). In brief, 1 g of sample was pulverized in liquid nitrogen, accurately weighed in a test tube, and mixed with 10 mL of acetonitrile solution and 8 μL of internal standard master batch. The extract was left overnight at 4 ℃, then centrifuged for 5 min at 12,000 g and 4 ℃, retaining the supernatant. The precipitates underwent two additional extractions with 5 mL of acetonitrile solution, and the resulting supernatants were combined. Impurities were removed using appropriate amounts of C18 and GCB, followed by centrifugation for 5 min at 12,000 g and 4 ℃. The supernatant was then dried under nitrogen, re-solubilized in 400 μL methanol, and filtered through a 0.22 μm organic phase membrane for subsequent LC–MS/MS analysis. Analytical conditions followed those described by Guo et al. (2021). MeSA and SA were quantified using standard curves ranging from 1 to 200 ng/mL.

### DAP-seq

The coding sequence (CDS) of WRKY40 was inserted into a vector containing an affinity tag, and a protein expression vector was constructed. Subsequently, in vitro protein expression was performed to generate a fusion protein comprising WRKY40 and the affinity tag. Genomic DNA extracted from apple leaves was used to construct a DNA library. The in vitro expressed WRKY40 fusion protein was then combined with the DNA library. Following this, the combined DNA was eluted and subjected to high-throughput sequencing. The resulting candidate downstream genes underwent KEGG clustering analysis (Bartlett et al. 2017).

### Yeast one-hybrid assays

Utilizing the ClonExpress II One-Step Cloning Kit (Cat. No. C112-01) from Vazyme, promoter fragments were inserted into the pAbAi vector, while the transcription factor's CDS sequence was incorporated into the pGADT7 vector. The primers employed for cloning are enumerated in Supplemental Table S2 and S3. The recombinant pAbAi vector was subsequently linearized using BstB1 enzyme, and the resulting digested product was introduced into Y1H yeast recipient cells, which were then cultured on SD/-ura medium. Positive monoclonal cell cultures underwent testing for self-activation on Aureobasidin A (ABA) selection medium to ascertain the ABA concentration that inhibits self-activation. The recombinant pGADT7 plasmid was then introduced into yeast recipient cells containing the pAbAi recombinant plasmid and cultured on SD/-Leu medium. Following the acquisition of positive monoclonal cell cultures, validation was conducted on medium supplemented with ABA to determine whether the transcription factor binds to the elements on the promoter.

### Electrophoretic mobility shift assay

The CDS was inserted into the pCOLD-TF vector. The resulting recombinant plasmid was subsequently introduced into Escherichia coli strain Rosetta (DE3) cells. The primers utilized for cloning are enumerated in Supplemental Table S2. The cells were cultivated in LB medium at 37 °C until the OD600 reached 0.6. The cell culture was then cooled to 16 °C before the addition of IPTG (0.30 mM final concentration) to induce protein expression. The recombinant protein was subsequently purified using glutathione beads. DNA oligos, procured from General Biosystems (Anhui) Co., Ltd, were labeled with biotin at the 5′ and 3′ ends. The DNA probe sequences are detailed in Supplemental Table S5. The DNA oligos were diluted with ddH2O and then combined with the purified protein for 60 min at 25 °C. Following incubation, the mixture underwent electrophoresis in a 6% native polyacrylamide gel using a 0.5 × Tris–borate-EDTA buffer for 1.5 h at 4 °C and 100 V. Prior to electrophoresis, the gel was flushed and preelectrophoresed for 60 min at 4 °C and 100 V. The biotin-labeled DNA in the gel was visualized using the ChemiDoc Imager (Bio-Rad).

### Dual-luciferase assay

The CDS were inserted into the pCAMBIA2300 overexpression vector (Hou et al. 2021). Promoter sequences were incorporated into the pLUC vector. Site-directed mutagenesis of the promoter sequences in the pLUC plasmid was performed using the Mut Express II Fast Mutagenesis Kit V2 (Vazyme, Item No. C214-01). Primers utilized for cloning are listed in Supplemental Tables S2 and S4. The recombinant pCAMBIA2300 and pLUC plasmids were introduced into EHA105 and EHA105 (pSoup) cells, respectively. *N. benthamiana* plants were co-transformed during their active growth phase, utilizing five fully expanded leaves per plant. The plants were cultivated under a 16 h light/8 h dark photoperiod at 30 °C for 48 h. LUC and Ren activity was quantified using the Dual Luciferase Reporter Assay Kit (Vazyme, Item No. DL101-01) or through in vivo imaging with Beyotime's D-Luciferin potassium salt (Item No. ST196).

### Yeast two-hybrid assays

Two CDS sequences were individually inserted into the pGBKT7 and pGADT7 vectors. Primers utilized for cloning are listed in Supplemental Tables S2. Recombinant pGBKT7 and pGADT7 plasmids functioned as negative controls. Co-transformation of AH109 recipient cells with recombinant pGBKT7 and pGADT7 plasmids was executed and subsequently plated on SD/-Leu-Trp medium. Following the identification of positive clones, protein–protein interactions were confirmed on SD/-Leu-Trp-Ade-His medium supplemented with 600 μM SA and 20 mg/L X-α-gal. pGBKT7-53 and pGADT7-T were employed as positive controls (Zhao et al. 2017).

### Yeast three-hybrid assays

To examine the effect of NPR3g on the protein–protein interaction between NPR1 and TGA2c-2, we employed a Y3H assay (Glass et al. 2015). The CDS of NPR1 was inserted into the pGBKT7 vector, while that of TGA2c-2 was cloned into the pGADT7 vector. The pGBKT7 vector was utilized to express the third protein, NPR3g. Primer sequences for cloning are provided in Supplemental Tables S2. The three recombinant plasmids were co-transformed into AH109 recipient cells and cultured on SD/-Leu-Trp medium. Colony PCR was used to confirm the presence of both NPR1 and NPR3g recombinant plasmids in individual clones. Negative controls consisted of monocultures containing the NPR1 recombinant plasmid and the empty pGBKT7 vector. Protein–protein interactions were verified on SD/-Leu-Trp-Ade-His medium supplemented with 600 μM SA and 20 mg/L X-α-gal.

### GST-pulldown

The two CDS sequences were individually cloned into the pGEX-4T-1 and pCOLD-TF vectors to express target proteins with GST and His tags, respectively. The primers utilized for cloning are listed in Supplemental Table S2. The recombinant plasmid was subsequently inserted into Escherichia coli strain Rosetta (DE3) cells. These cells were cultured in LB medium at 37 °C until the OD600 reached 0.6. The cell culture was then cooled to 16 °C before IPTG was added (0.30 mM final concentration) to induce protein expression. The recombinant protein was purified using glutathione beads. After purification of the GST- and His-tagged target proteins, they were combined in a 1:1 ratio and incubated at 4°C for 3 h. During this incubation period, SA was introduced to the protein solution to achieve a final concentration of 600 μM. Following incubation, a GST-pulldown assay was conducted, and the filtrate was collected as the eluate for Western Blotting.

### Bimolecular fluorescence complementation

The CDS sequences of two genes were individually cloned into the pYFPn and pYFPc vectors. The primers used for cloning are listed in Supplemental Table S2. The recombinant plasmids were transformed into EHA105 competent cells. Agrobacterium was cultured at 28°C with agitation until reaching an OD of approximately 0.5. Subsequently, the two bacterial cultures were combined in a 1:1 ratio and introduced into tobacco cells. Following application of 0.4 mM SA, the plants were maintained under standard conditions at 30 °C with a 16h light/8h dark photoperiod for 2 days. Yellow fluorescent protein in tobacco cells was visualized using a confocal laser scanning microscope. Empty pYFPn and pYFPc vectors served as a negative control, while MdMYB308L-pYFPn and MdbHLH33-pYFPc combinations functioned as positive controls (An et al. 2019).

### Plant genetic transformation and characterization

The modified binary vector pCAMBIA2300 was redesignated as pCAMBIA2300-GUS. Its structural schematic is presented in Supplemental Fig. 18. All subsequent plasmids employed for stable genetic transformation utilized pCAMBIA2300-GUS as the backbone. The CDS sequence of the target gene was inserted into pCAMBIA2300-GUS. The primers used for cloning are listed in Supplemental Table S2. For apple genetic transformation methods, refer to Hou et al. (2021).

This study employed a simplified method for constructing RNAi vectors. The pRNAi-E vector was generated by inserting an intron sequence, derived from the pKANNIBAL vector, into the polyclonal site of the pCAMBIA2300-GUS vector; the intron sequence is presented in Supplemental Fig. 20. Subsequently, the RNAi vector for the target gene was constructed by ligating the forward and reverse fragments of the gene-specific sequences to both sides of the intron, respectively (Song et al. 2017). A structural schematic of this process is illustrated in Supplemental Fig. 19. The specific fragments of WRKY1, WRKY40, and NPR3g used for constructing the RNAi vector are detailed in Supplemental Fig. 21. GUS staining was employed to confirm successful transformation, followed by an RT-qPCR assay to validate the transformation efficiency.

### RT-qPCR analysis

Quantitative reverse transcription PCR (RT-qPCR) experiments were conducted using the QuantStudio 5 instrument. The gene identifiers and primers utilized in this study are presented in Supplemental Table S1.

### Trypan blue stain

The leaf samples were immersed in trypan blue staining solution and placed in a boiling water bath for 2 min, then left at room temperature overnight. Subsequently, the leaves were removed and decolorized in chloral decolorizing solution for three days, with the solution being replaced daily. Following decolorization, the spores and mycelium were examined using a super-field-depth stereomicroscope. The trypan blue staining solution (100 mL) was prepared using 50 mg of trypan blue, 25 mL of lactic acid, 23 mL of water-soluble phenol (7%), 25 mL of glycerol, and 27 mL of water. The decolorizing solution consisted of 100% chloral solution (Zhang et al. 2018).

### Subcellular localization

The CDS of *WRKY40* and *WRKY1* were individually inserted into the pCAMBIA1302 vector. The primers utilized for cloning are enumerated in Supplemental Table S2. The recombinant plasmids were subsequently introduced into EHA105 recipient cells. Agrobacterium cultures were incubated at 28°C with agitation until reaching an optical density (OD) of approximately 0.5, after which they were injected into tobacco cells. The plants were then cultivated under standard conditions at 30 °C, with a 16h light/8h dark photoperiod for 2 days. Green fluorescent protein in tobacco cells was visualized using a confocal laser scanning microscope. DAPI staining served as a nuclear localization marker (Yao et al. 2019).

### Conservative domain analysis

*Arabidopsis thaliana* gene sequences were obtained from TAIR, while apple genome files were downloaded from GDR (Daccord et al. 2017). The protein sequences of *Arabidopsis* genes were compared to the apple genome to determine corresponding apple gene IDs utilizing the Blast plugin in TBtools (Chen et al. 2020). Conserved domain analysis was conducted using the Batch CD-search tool available on NCBI.

## Supplementary Information

Supplementary Material 1. Supplementary figures that substantiate the findings of this study.

Supplementary Material 2. Primers used in this research.

## Data Availability

The datasets generated and/or analyzed during the current study are available from the corresponding author upon reasonable request.

## Abbreviations

PM: Powdery mildew
SA: Salicylic acid
PAL: Phenylalanine ammonia lyase
ICS: Isochorismate synthase
DAP-seq: DNA affinity purification sequencing
TTS: Transcription termination site
EMSA: Electrophoretic mobility shift assays
Y1H: Yeast one-hybrid
LUC: Luciferase
Y2H: Yeast two-hybrid assays
Y3H: Yeast three-hybrid assays
BiFc: Bimolecular fluorescence complementation
PR1: Pathogenesis-related 1
ABA: Aureobasidin A
