# Supplementary material for: A novel mode of WRKY1 regulating PR1-mediated immune balance to defend against powdery mildew in apple
Source: Mol Hortic. 2025 Mar 5;5:17. doi: 10.1186/s43897-024-00141-z (PMC11881497; doi:10.1186/s43897-024-00141-z)
Supplement: Supplementary file 1 — Supplementary Material 1. Supplementary figures that substantiate the findings of this study. [file 43897_2024_141_MOESM1_ESM.docx]

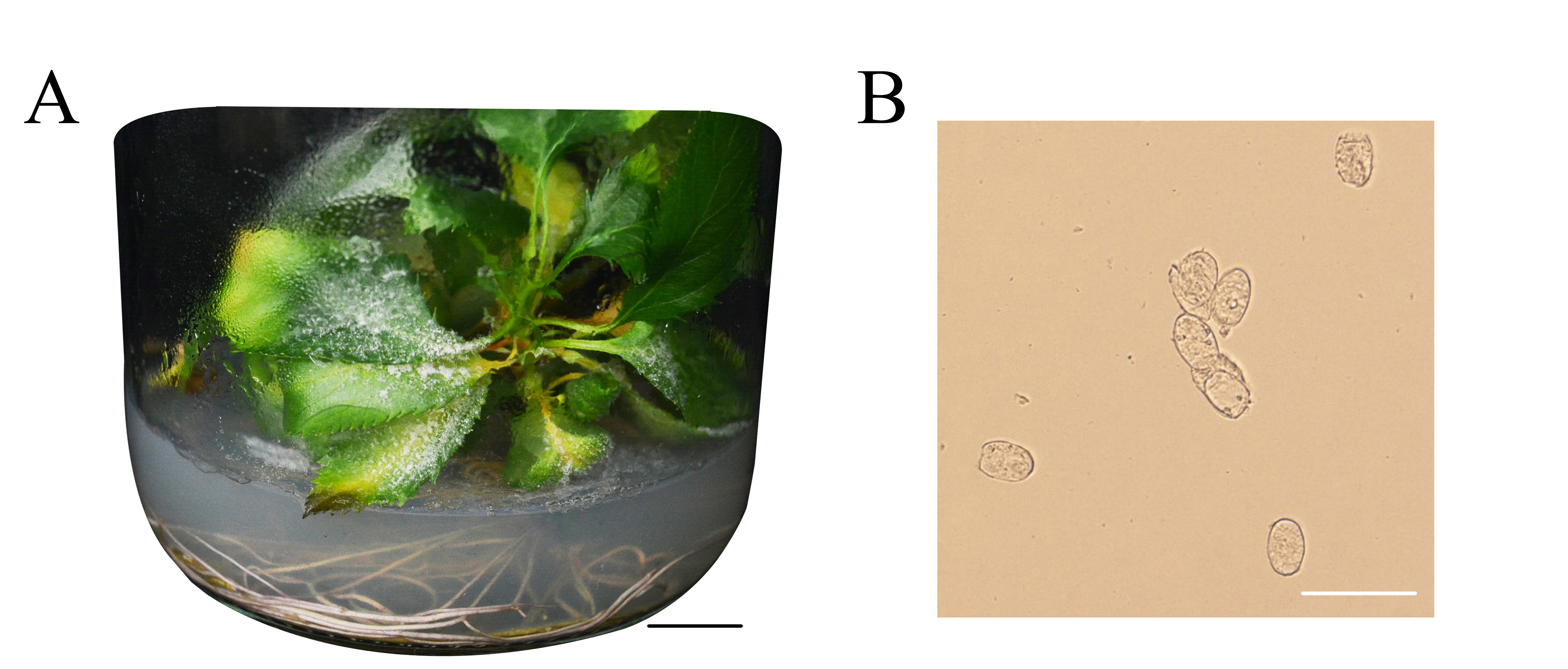


Supplemental Figure 1. Preservation of Powdery mildew (PM) and microscopic observation of spores. (A) PM is inoculated onto rooted tissue culture seedlings. (B) PM spores are observed under an optical microscope. The black and white scales represent 1 cm and 50 μm, respectively.


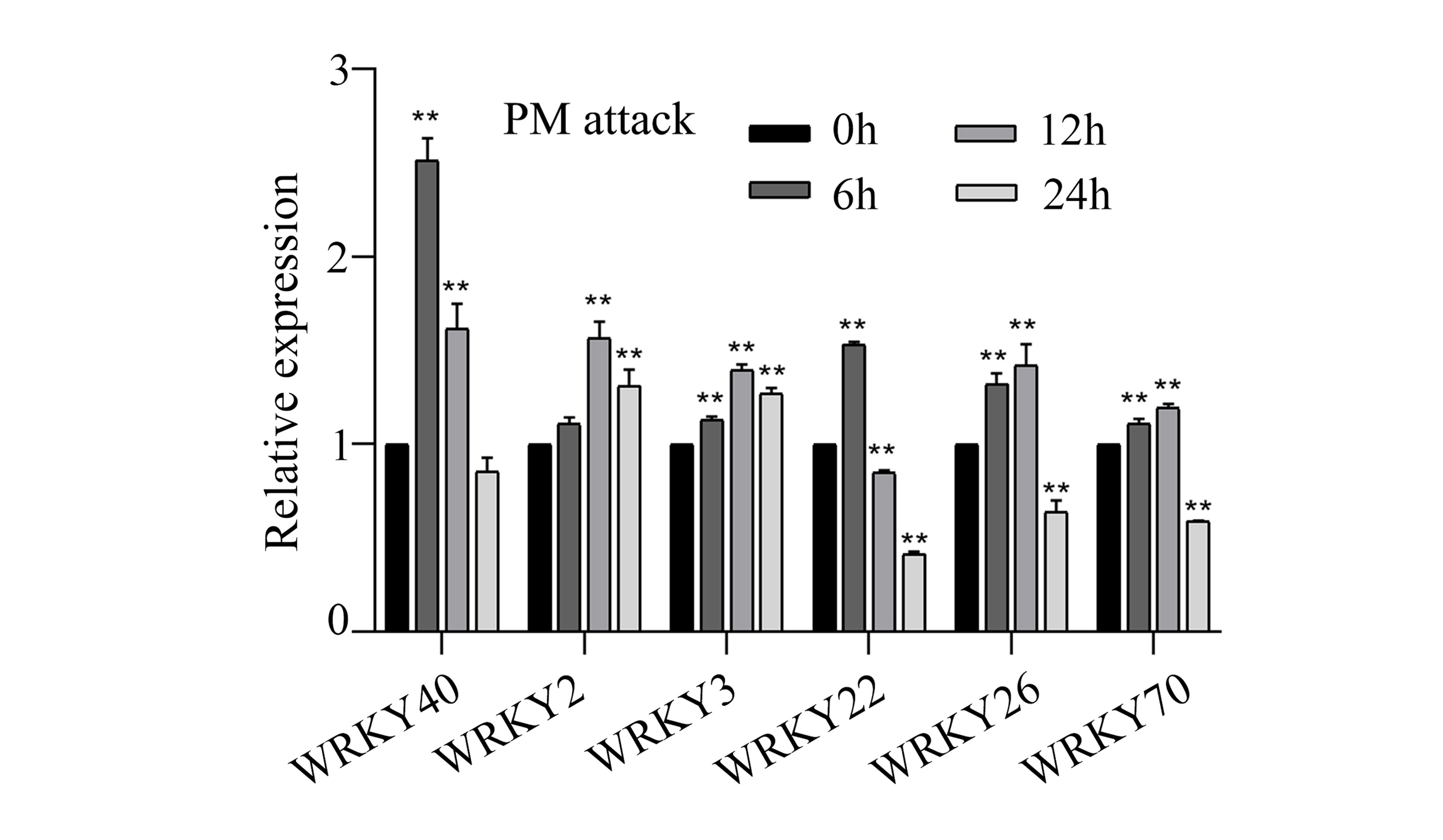


Supplemental Figure 2. The RT-qPCR showed the expression of six WRKY transcription factors in wild type (WT) plants after PM attack. The data represent the means and standard deviations of three independent replicate experiments. Asterisks (*) indicate significant differences from the control (Student’s t test, **P < 0.01).


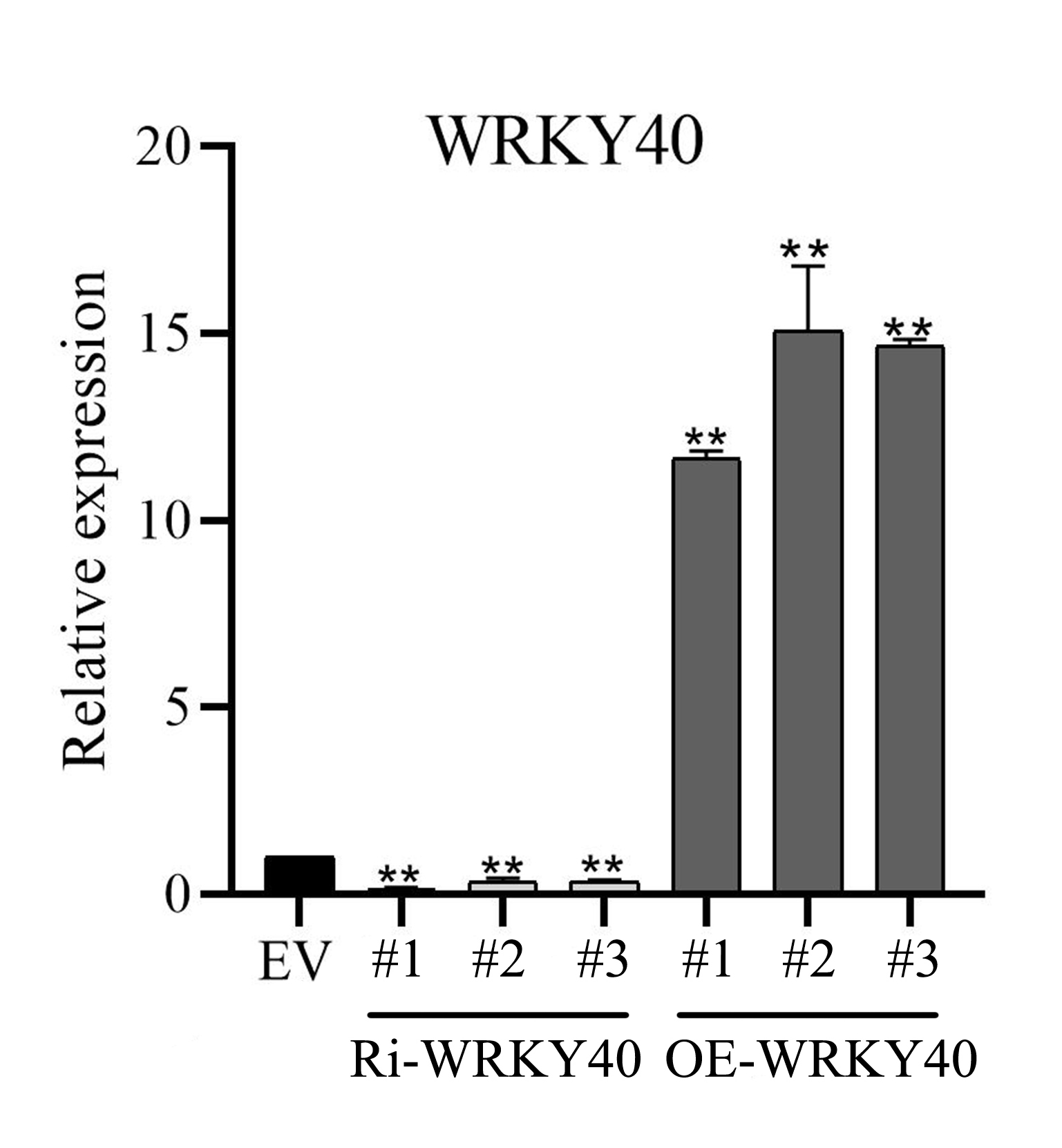


Supplemental Figure 3. Expression levels of *WRKY40* in *WRKY40* transgenic plants. Ri-*WRKY40* represents RNAi-silenced *WRKY40* plants, OE-*WRKY40* represents *WRKY40*-overexpressing plants, and EV represents empty vector control plants. The data represent the means and standard deviations of three independent replicate experiments. Asterisks (*) indicate significant differences from the control (Student’s t test, **P < 0.01).


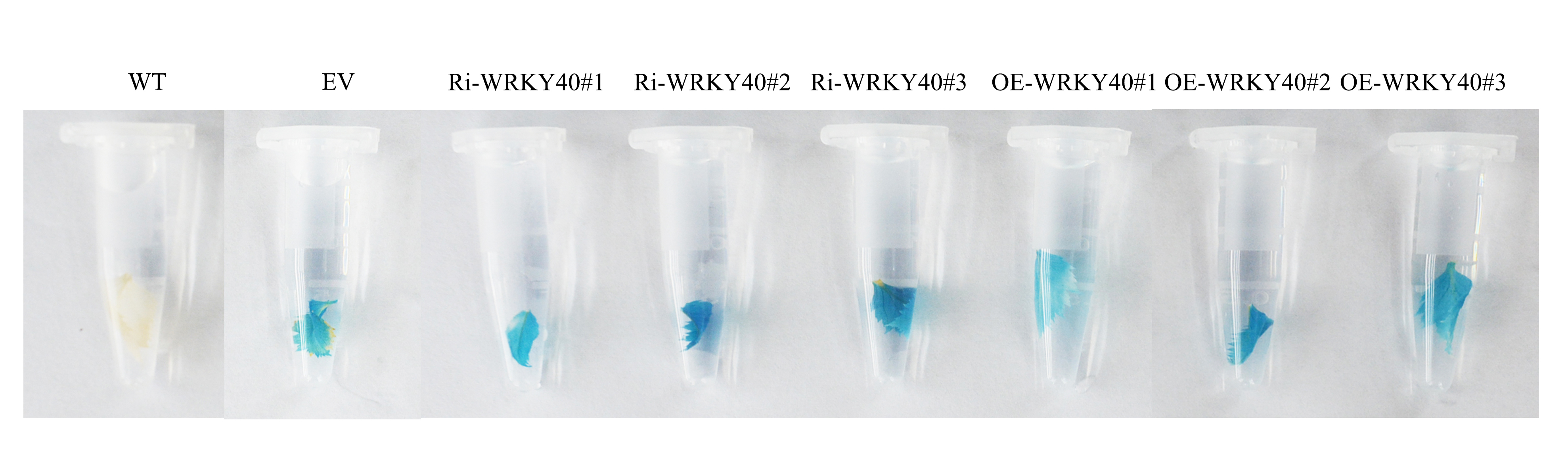


Supplemental Figure 4. GUS staining of Silencing and overexpression plants of *WRKY40*. Ri-*WRKY40* represents RNAi-silenced *WRKY40* plants, OE-*WRKY40* represents *WRKY40*-overexpressing plants, EV represents empty vector control plants, and WT represents wild type plants.


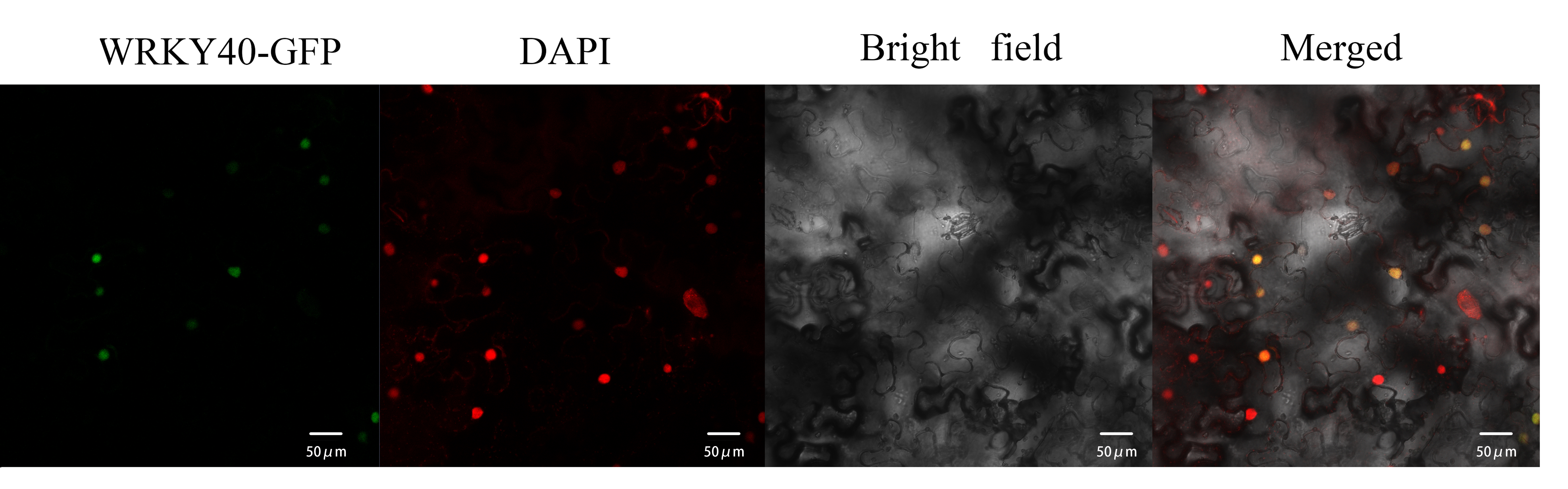


Supplemental Figure 5. Subcellular localization of WRKY40. The nucleus localization of WRKY40 in *Nicotiana benthamiana*. The green fluorescence and the red fluorescence were, respectively, from the WRKY40-GFP, and the nucleus fluorescent probe (DAPI). Scale bar is 50 μm.


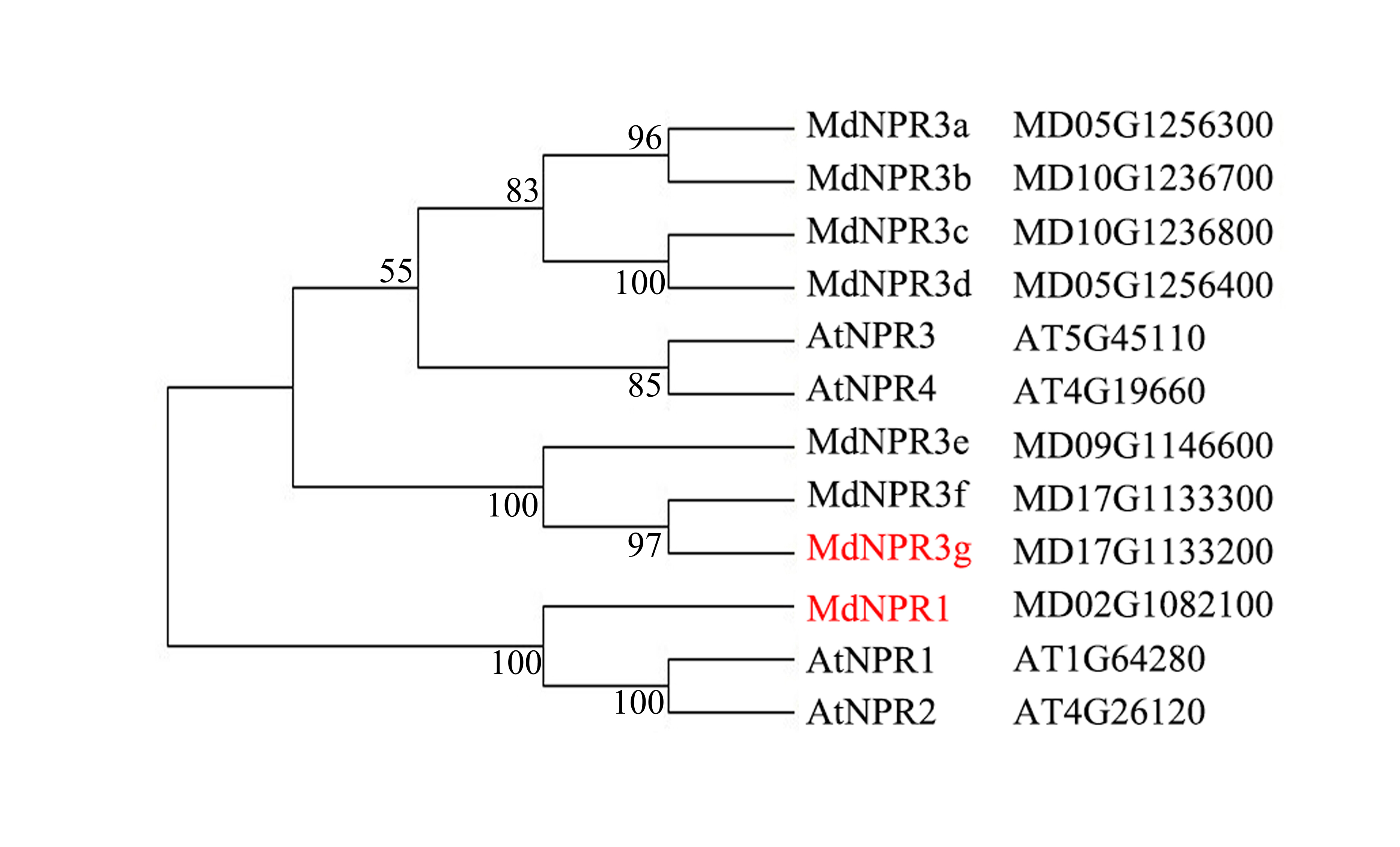


Supplemental Figure 6. Phylogenetic tree showed parental relationship between all NPR-like genes (renamed as NPR3a-g), NPR1 and Arabidopsis AtNPR1/2/3/4.


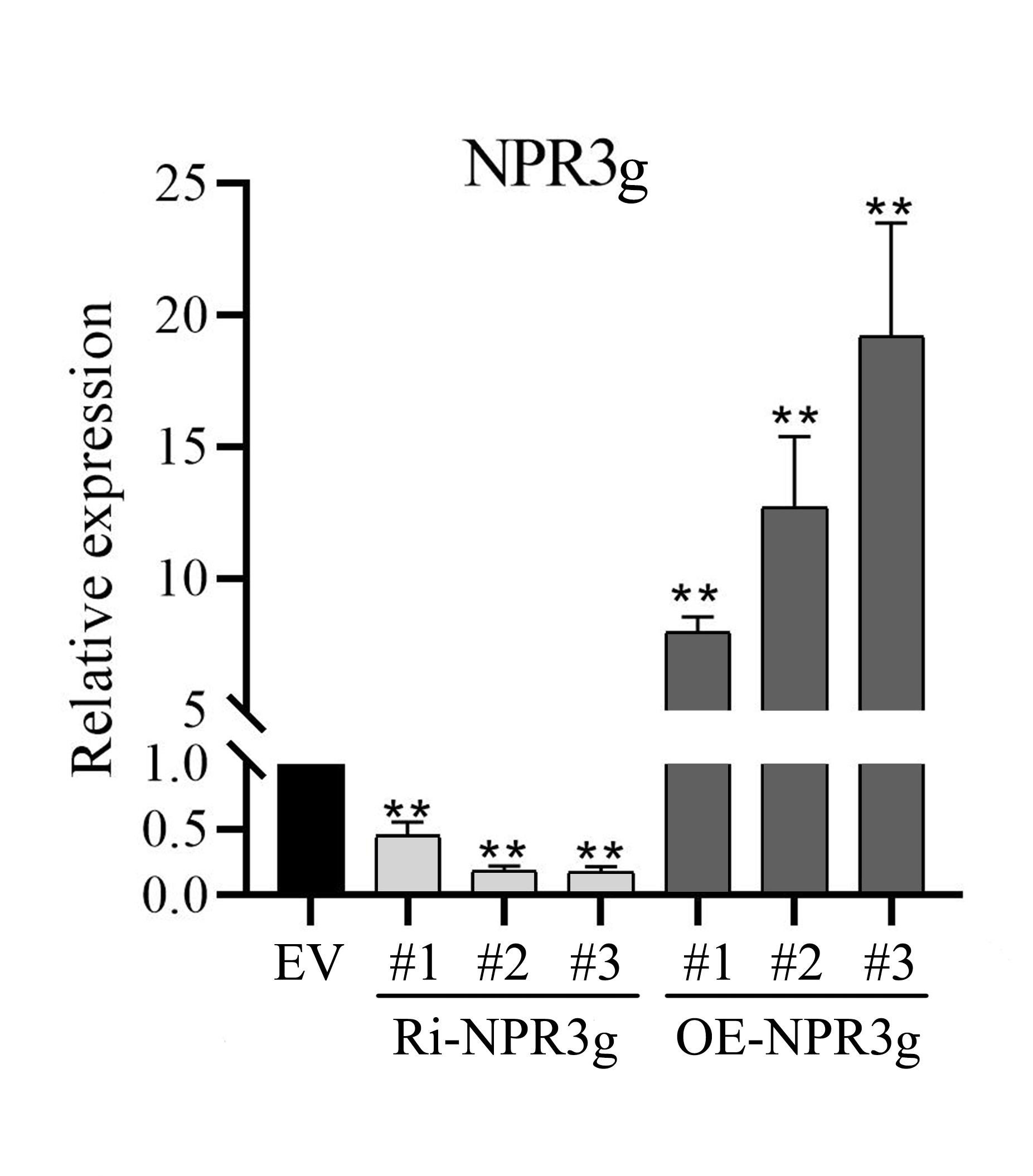


Supplemental Figure 7. Expression levels of *NPR3g* in *NPR3g* transgenic plants. Ri-*NPR3g* represents RNAi-silenced *NPR3g* plants, OE-*NPR3g* represents *NPR3g*-overexpressing plants, and EV represents empty vector control plants. The data represent the means and standard deviations of three independent replicate experiments. Asterisks (*) indicate significant differences from the control (Student’s t test, **P < 0.01).


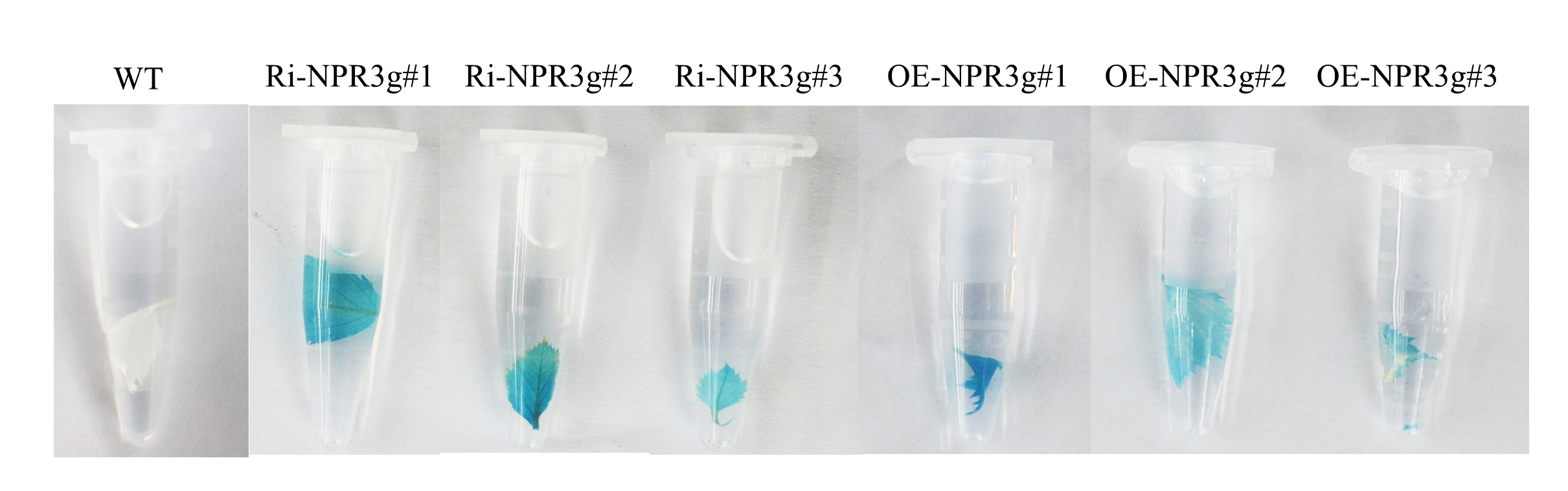


Supplemental Figure 8. GUS staining of Silencing and overexpression plants of *NPR3g*. Ri-*NPR3g* represents RNAi-silenced *NPR3g* plants, OE-*NPR3g* represents *NPR3g*-overexpressing plants, and WT represents wild type plants.


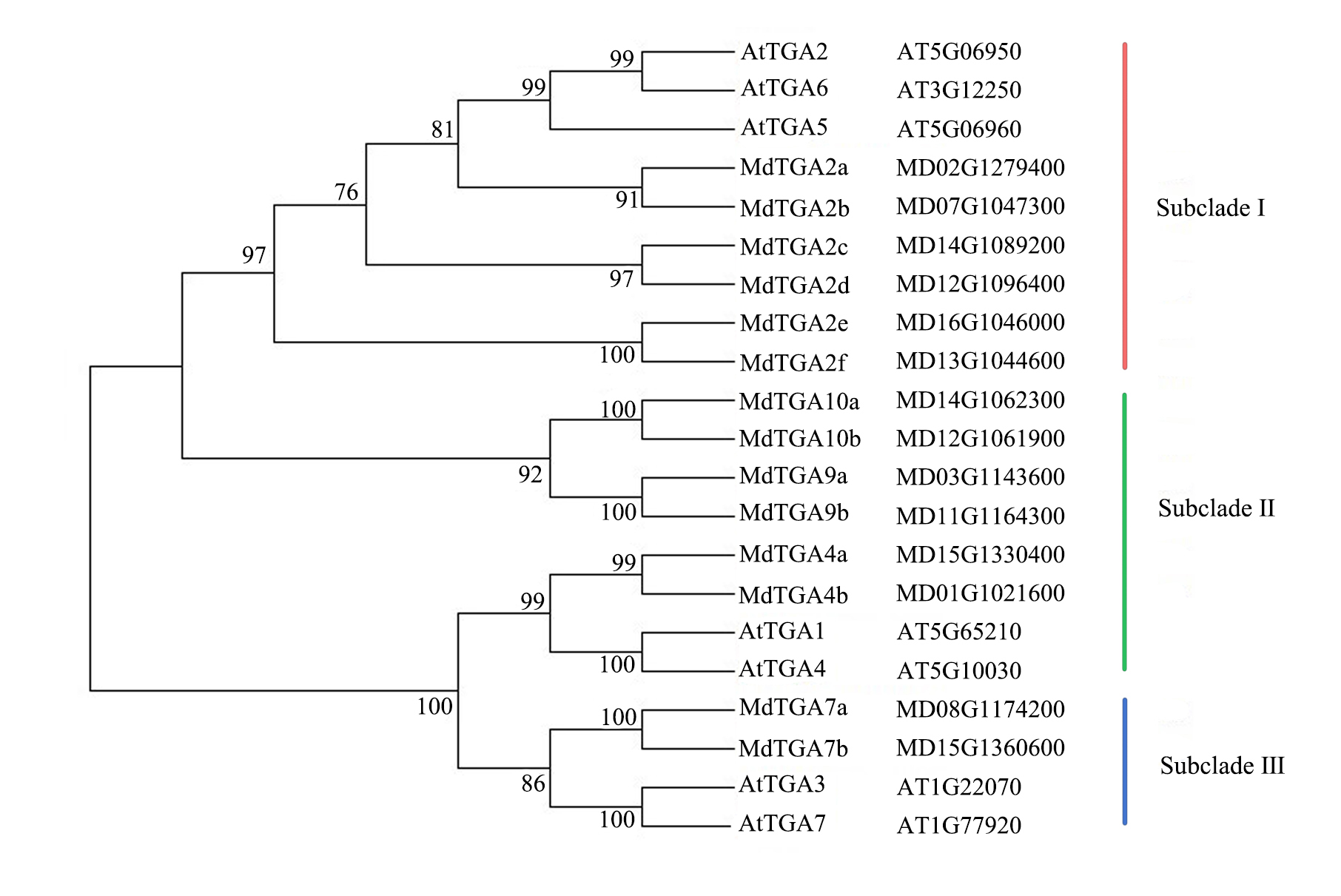


Supplemental Figure 9. Phylogenetic tree showed parental relationship between all TGAs genes and *Arabidopsis* AtTGA1-7. The genes are clustered into three subclades.


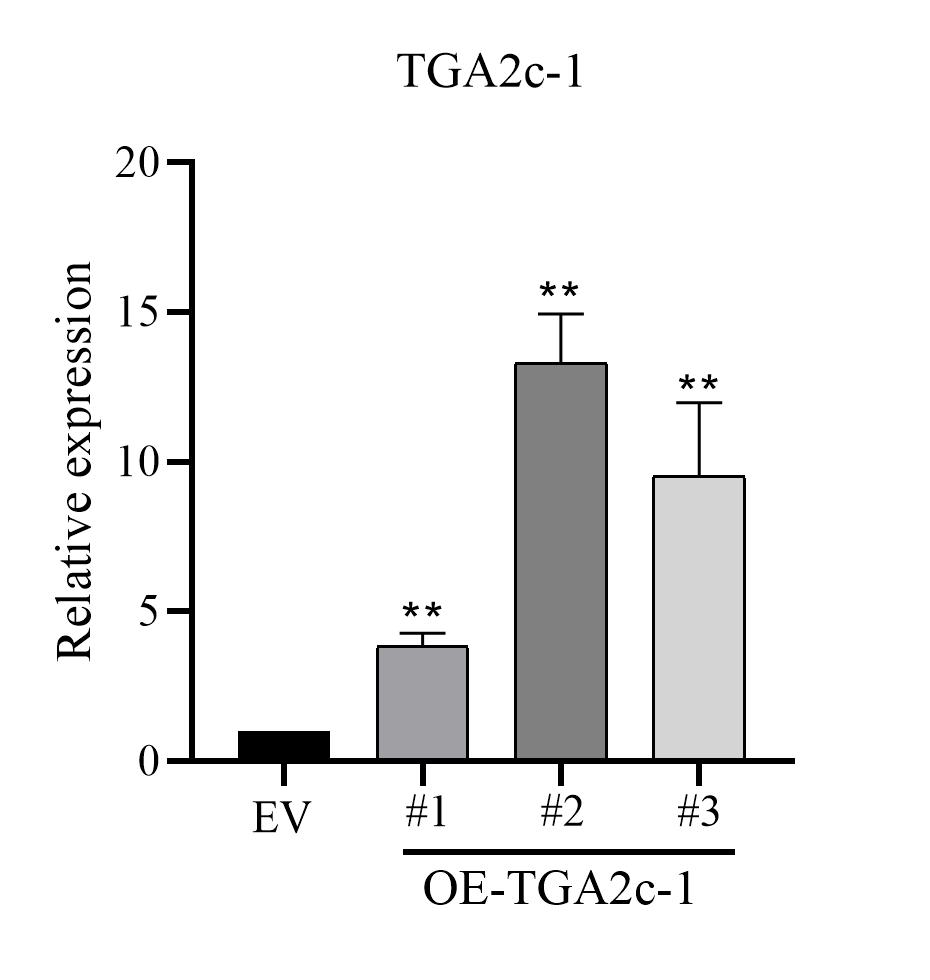

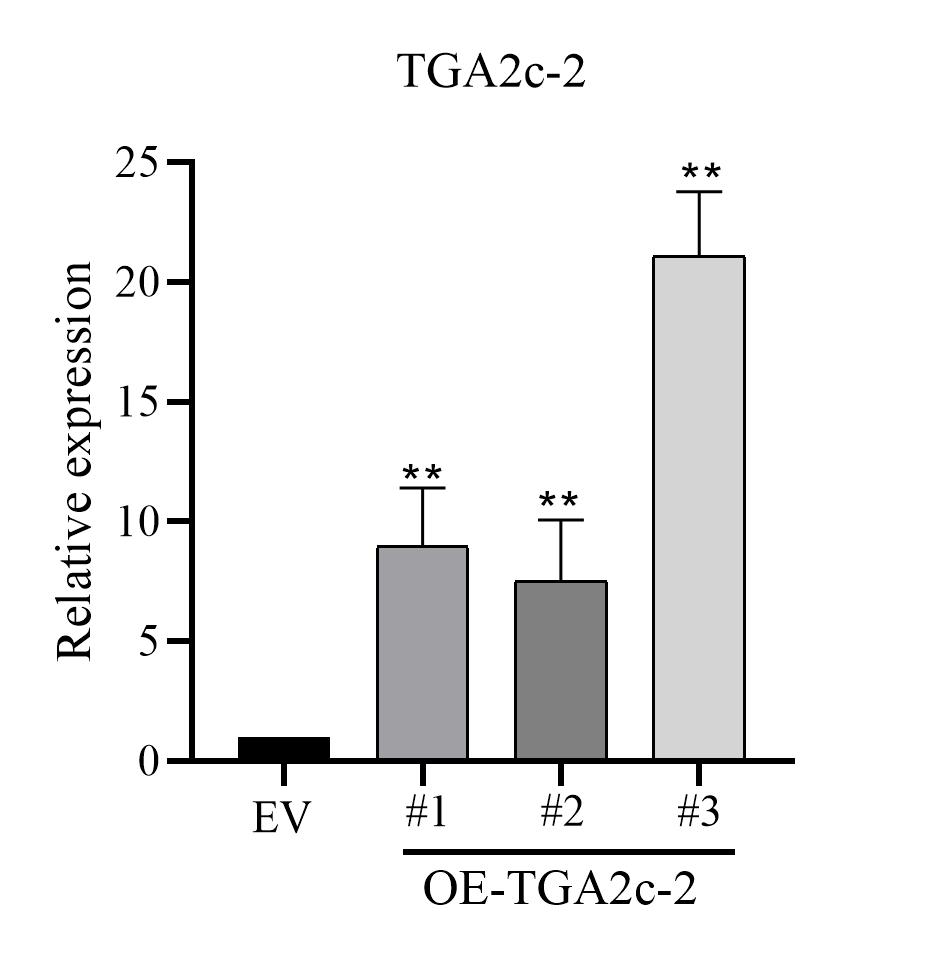


Supplemental Figure 10. The RT-qPCR showed the expression of TGA2C-1/2 in TGA2C-1/2 over-expressed lines. The data represent the means and standard deviations of three independent replicate experiments. Asterisks (*) indicate significant differences from the control (Student’s t test, **P < 0.01).


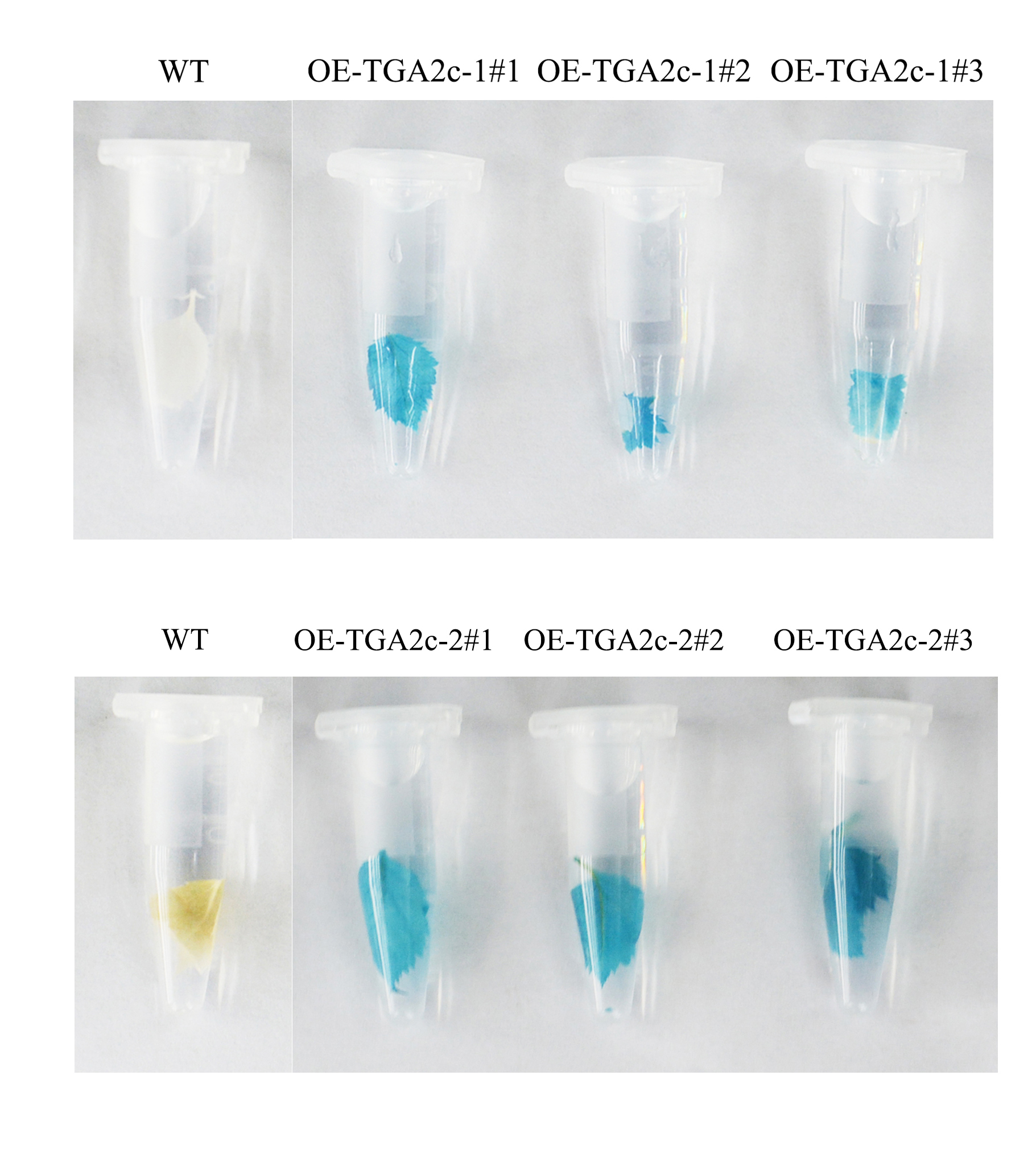


Supplemental Figure 11. GUS staining of overexpression plants of *TGA2c-1/2*. OE-*TGA2c-1/2* represents *TGA2c-1/2* overexpressed plants, and WT represents wild-type plants.


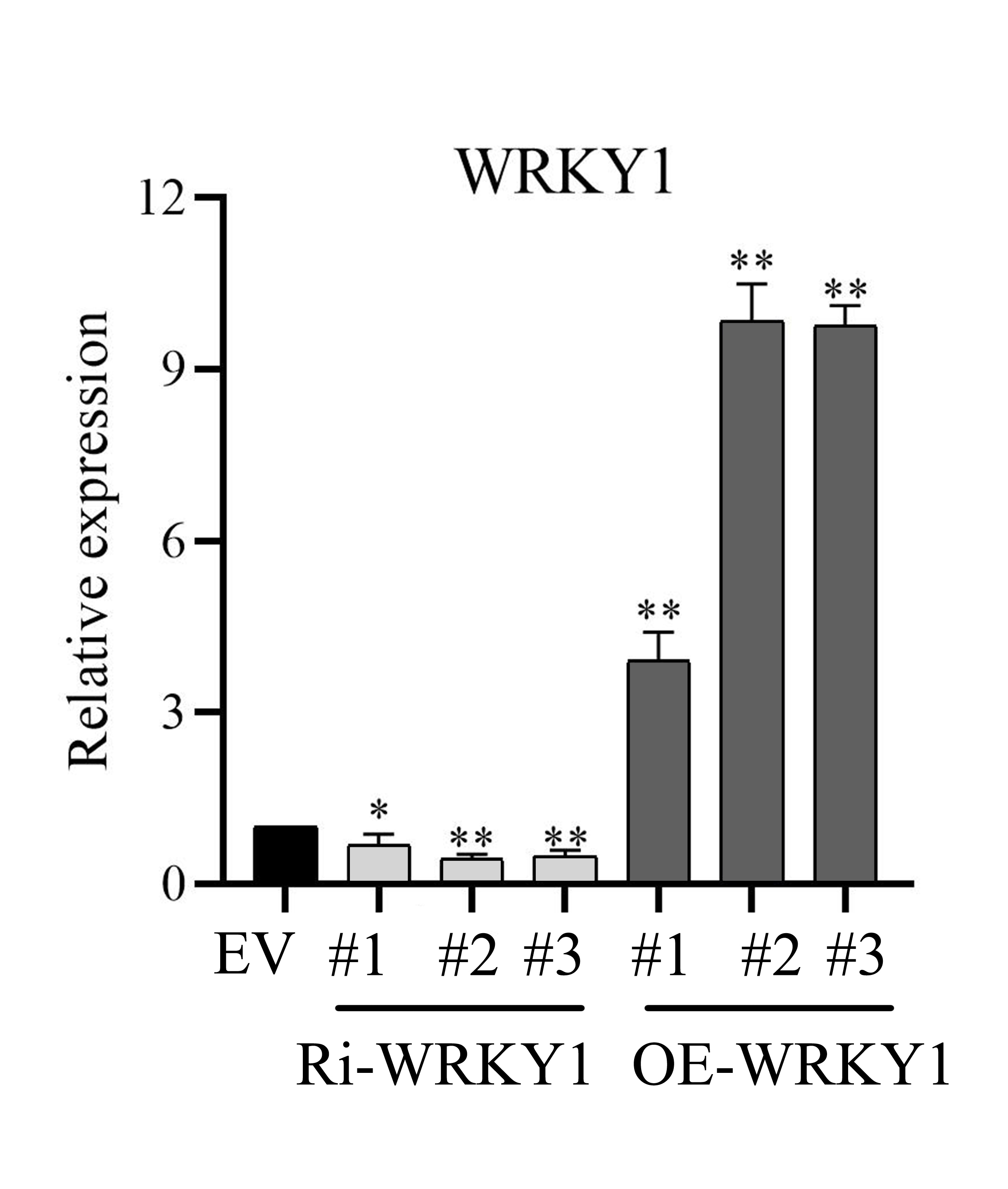


Supplemental Figure 12. Expression levels of *WRKY1* in *WRKY1* transgenic plants. Ri-*WRKY1* represents RNAi-silenced *WRKY1* plants, OE-*WRKY1* represents *WRKY1*-overexpressing plants, and EV represents empty vector control plants. The data represent the means and standard deviations of three independent replicate experiments. Asterisks (*) indicate significant differences from the control (Student’s t test, **P < 0.01).


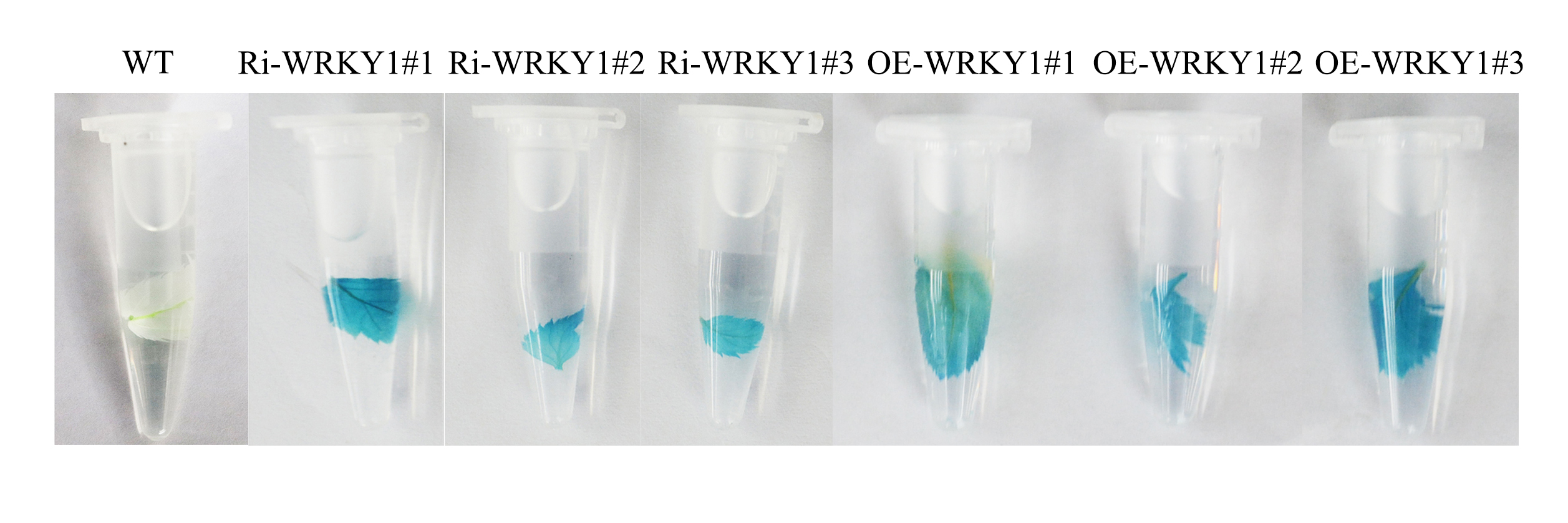


Supplemental Figure 13. GUS staining of Silencing and overexpression plants of *WRKY1*. Ri-*WRKY1* represents RNAi-silenced *WRKY1* plants, OE-*WRKY1* represents *WRKY1*-overexpressing plants, and WT represents wild type plants.


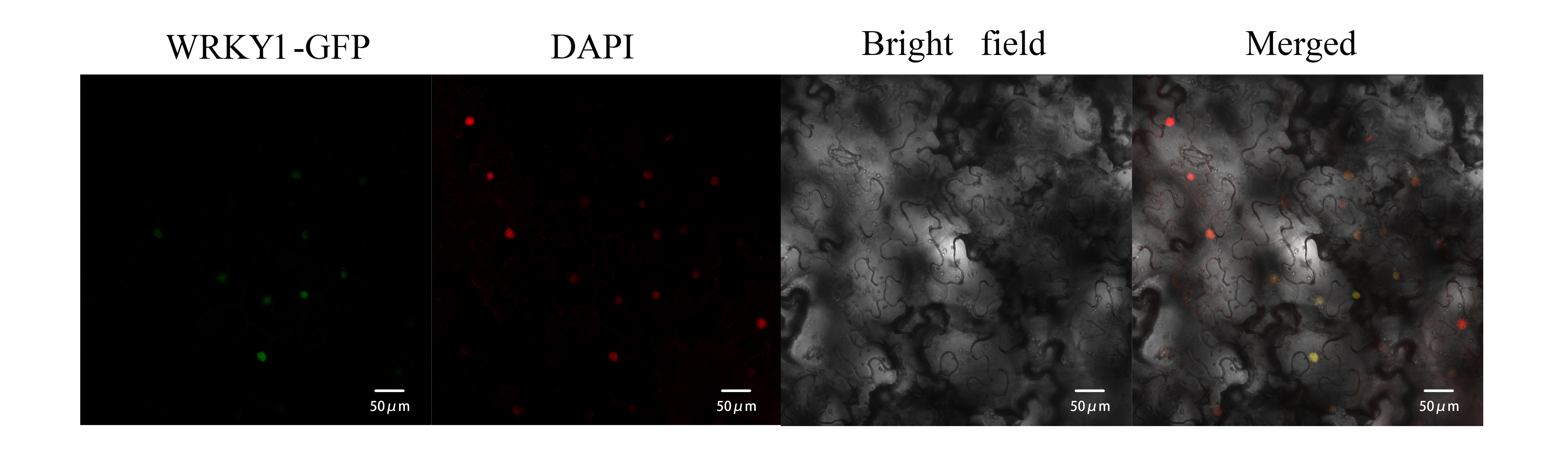


Supplemental Figure 14. Subcellular localization of WRKY1. The nucleus localization of WRKY1 in *Nicotiana benthamiana*. The green fluorescence and the red fluorescence were, respectively, from the WRKY1-GFP, and the nucleus fluorescent probe (DAPI). Scale bar is 50 μm.


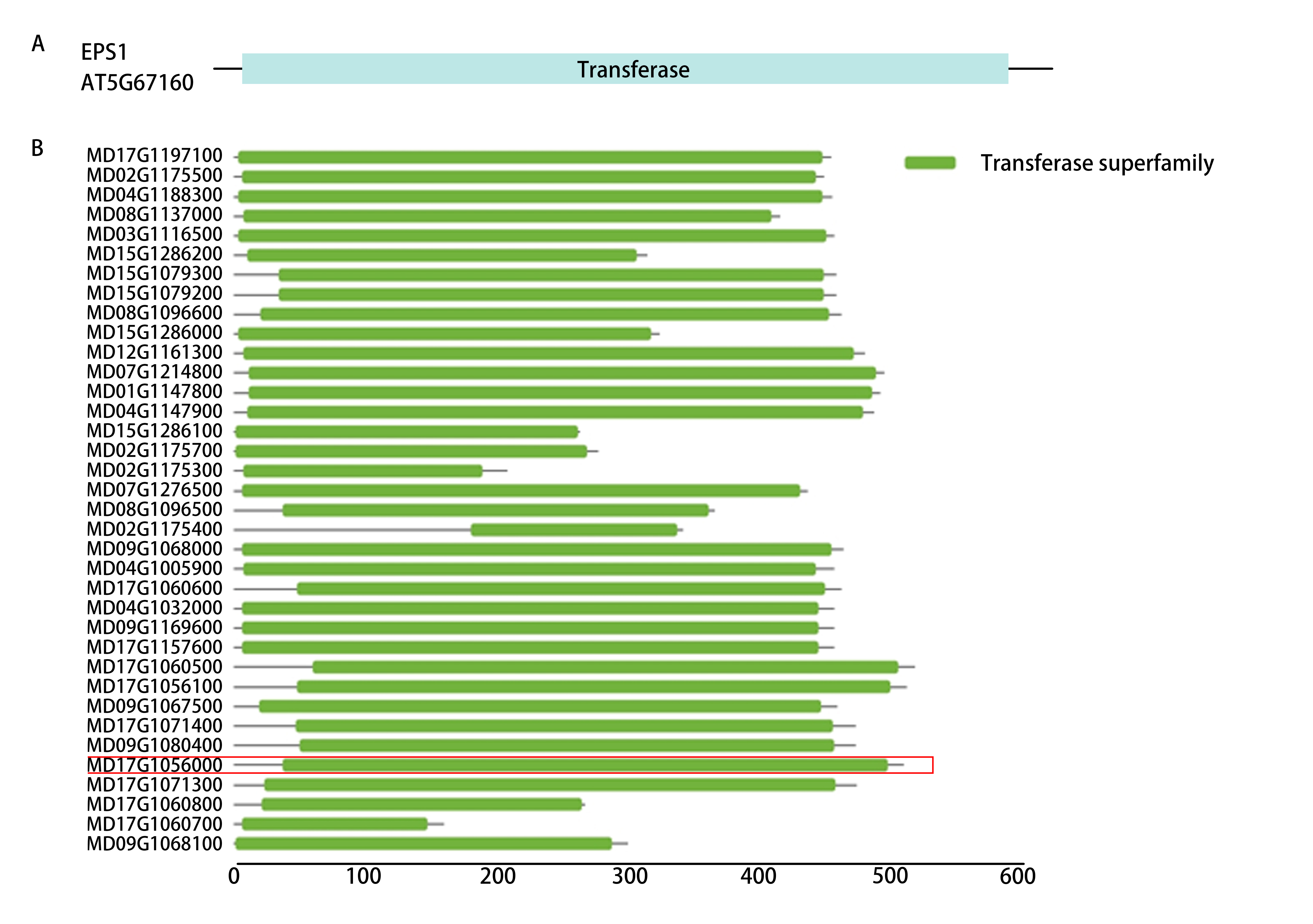


Supplemental Figure 15. (A) Domain of *Arabidopsis* At*EPS1*. (B) The domains of the 36 apple *EPS1-like* genes were obtained. The gene in the red box (MD17G1056000) is a direct downstream gene of WRKY1.


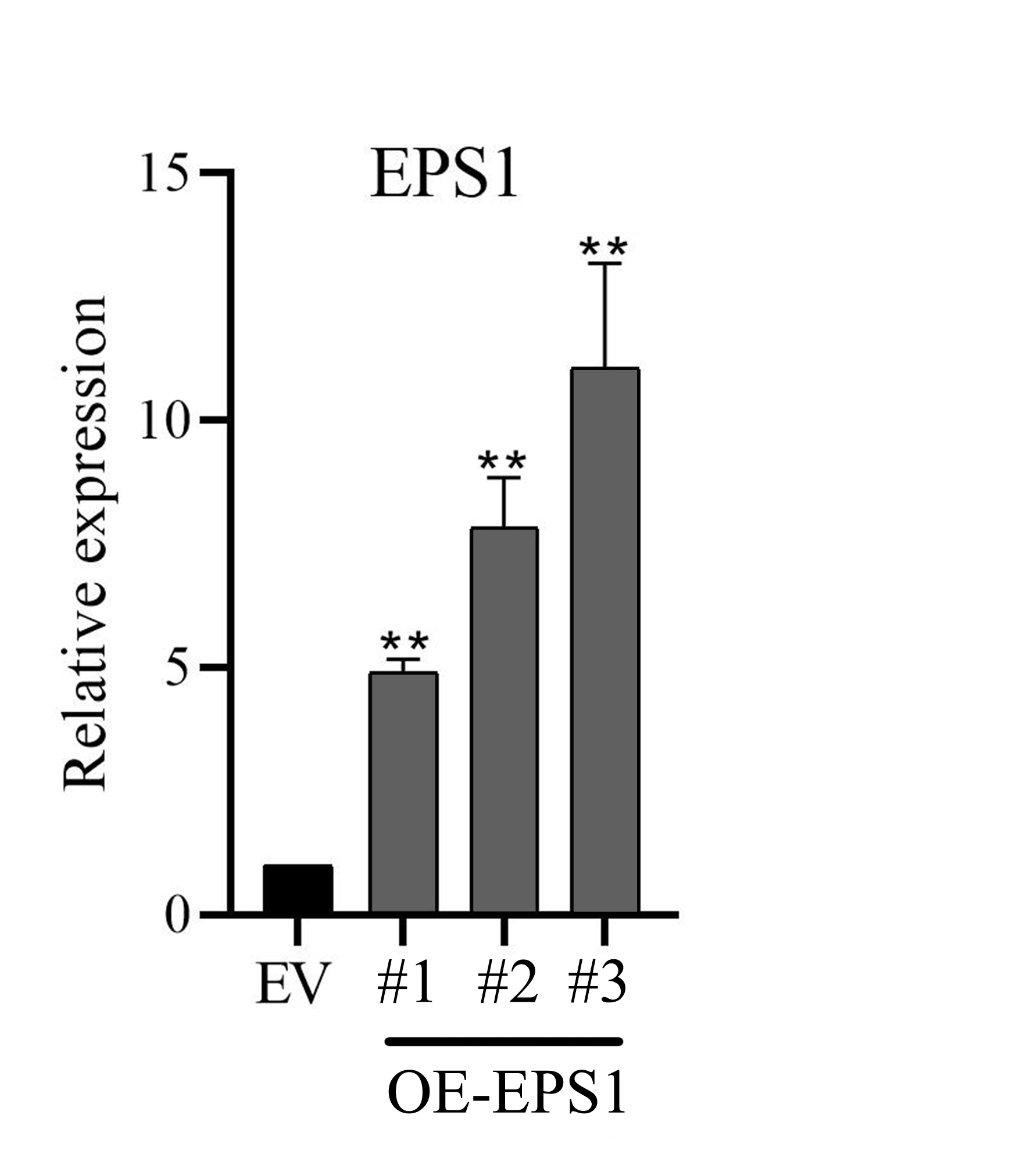


Supplemental Figure 16. The RT-qPCR showed the expression of *EPS1* in *EPS1* over-expressed lines. The data represent the means and standard deviations of three independent replicate experiments. Asterisks (*) indicate significant differences from the control (Student’s t test, **P < 0.01).


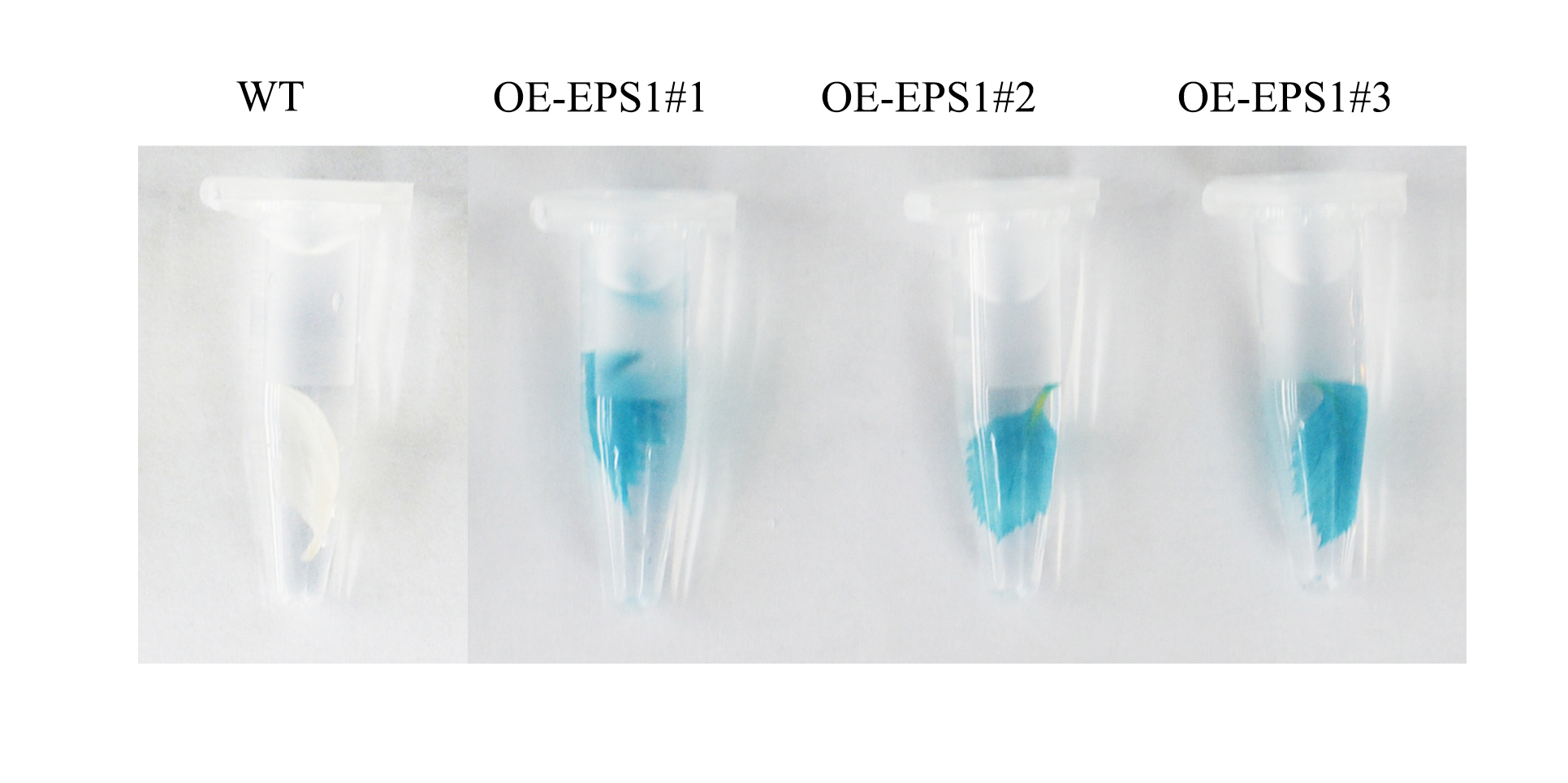


Supplemental Figure 17. GUS staining of overexpression plants of *EPS1*.


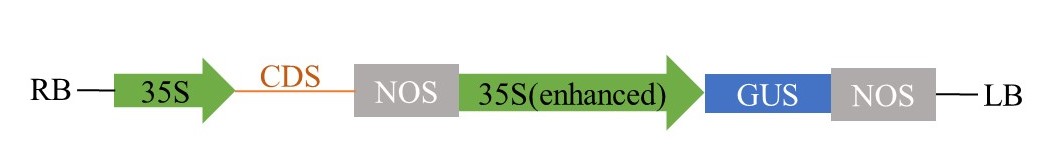


Supplemental Figure 18. The structure of pCAMBIA2300-GUS is shown, which is a modified pCAMBIA2300 vector. CDS is coding sequence of target genes.


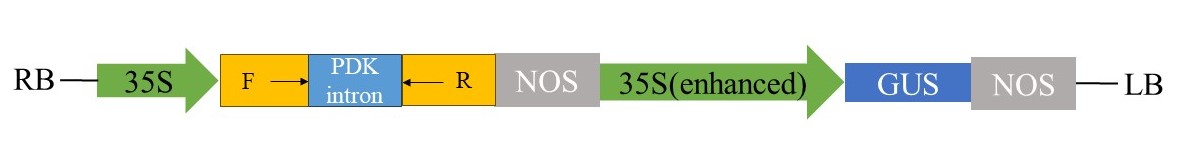


Supplemental Figure 19. The structure of RNAi vector is shown, which is constructed using pCAMBIA2300-GUS as the destination vector. The PDK intron is intron sequence derived from the pKANNIBAL vector. The F and R is forward and reverse complementary fragments of the target gene-specific sequences, respectively.

CGCTCGAGGAATTCGGTACCCCAATTGGTAAGGAAATAATTATTTTCTTTTTTCCTTTTAGTATAAAATAGTTAAGTGATGTTAATTAGTATGATTATAATAATATAGTTGTTATAATTGTGAAAAAATAATTTATAAATATATTGTTTACATAAACAACATAGTAATGTAAAAAAATATGACAAGTGATGTGTAAGACGAAGAAGATAAAAGTTGAGAGTAAGTATATTATTTTTAATGAATTTGATCGAACATGTAAGATGATATACTAGCATTAATATTTGTTTTAATCATAATAGTAATTCTAGCTGGTTTGATGAATTAAATATCAATGATAAAATACTATAGTAAAAATAAGAATAAATAAATTAAAATAATATTTTTTTATGATTAATAGTTTATTATATAATTAAATATCTATACCATTACTAAATATTTTAGTTTAAAAGTTAATAAATATTTTGTTAGAAATTCCAATCTGCTTGTAATTTATCAATAAACAAAATATTAAATAACAAGCTAAAGTAACAAATAATATCAAACTAATAGAAACAGTAATCTAATGTAACAAAACATAATCTAATGCTAATATAACAAAGCGCAAGATCTATCATTTTATATAGTATTATTTTCAATCAACATTCTTATTAATTTCTAAATAATACTTGTAGTTTTATTAACTTCTAAATGGATTGACTATTAATTAAATGAATTAGTCGAACATGAATAAACAAGGTAACATGATAGATCATGTCATTGTGTTATCATTGATCTTACATTTGGATTGATTACAGTTGGGAAATTGGGTTCGAAATCGATAAGCTTGGATCCTCTAGAGTC

Supplemental Figure 20. The intron sequence is derived from the pKANNIBAL vector.

WRKY1

GTTGCTGGCTCAGATATGGTCGTTGATTCTGATCTAGGCTCCGAAAGAACACTACATGATCAATCGGTTGCCGAAGCATGTACGACAACGGAAAGCGACCCCCCTTACATGATTGTTTGCAGAGCAAATCATAAGAAAACTATTGAGATGGGAATTAAGTCAGAAGGCGTTGATATGGTGGCTTGCGACAATCTGCGTCCGGAAAGTAAT

WRKY40

AGTGATGATAAAGAGTATTCATGCAAGAGGCTAAAGGAGAACACCAACTTAAAGATTTCCAGGGTTTATGTTCACACCGATGCATCCGATACAGGCCTCATTGTGAATGATGGATACCAATGGAGGAAATATGGTCAGAAGGTCACAAGAGATAACCCATCTCCTAGGGCTTACTACAAGTGCTCCTTTGCCCCAAGTTGCCCTGTTAAAAAGAAGGTGCAAAAAAGTGCTGAAAATCCATGCCTACTGGTGGCTACATATGAAGGAGAACACAACCACATGTATCCTGAGAAGCGAGCTGAAGTTACACTAATAGCCCCGGTATCTCCAAAACAGAACCAGCAGCTTATT

NPR3g

TCGGCCAGTTCTCAAAGTATTGAACATTATTTGAGTCTAAGCAAACTGAGTGATAATCTTGAGAGACTGTTACTTGATCATGAGTATGACTATAGTGATGCTGAGATTGTTGTTGAGAGAAACACCGTGGGTGTCAATCGGTGTATATTGGCTTCCCGGAGTCAGTTTTTTCAGTACCTTTTTAGGAAGGGGAGTGATGATGCGAAGACGGAAGGTAAACCGAGGTATCTCATGTCTGAATTGGTTCCAAACG

Supplemental Figure 21. The specific fragments of WRKY1, WRKY40, and NPR3g for constructing RNAi vectors.
