# Supplementary material for: A novel mode of WRKY1 regulating PR1-mediated immune balance to defend against powdery mildew in apple
Source: Mol Hortic. 2025 Mar 5;5:17. doi: 10.1186/s43897-024-00141-z (PMC11881497; doi:10.1186/s43897-024-00141-z)
Supplement: Supplementary file 2 — Supplementary Material 2. Primers used in this research. [file 43897_2024_141_MOESM2_ESM.docx]

Supplemental table S1 Gene IDs and primers for RT-qPCR in this study

| Gene ID | Gene name | F | R |
| --- | --- | --- | --- |
| MD00G1143500 | WRKY40 | GATGGATACCAATGGAGGAA | GCTCGCTTCTCAGGATAC |
| MD03G1044400 | WRKY2 | TCTGGTGAGGATATAACTGAAG | GTGTGGTTGGACTGAGAC |
| MD16G1066500 | WRKY3 | ACTCTCGTCTCCAACTTCT | CGCTCTGTTCTGCTTGAA |
| MD01G1071300 | WRKY22 | AACAGCAACTCCTCTTCTC | TTGTTGTTGTTGGTGTATGG |
| MD03G1057400 | WRKY26 | ATGGATACAGATGGAGGAAGTA | ACTGCTCTAATATCGTGAGAAG |
| MD01G1168600 | WRKY70 | GACTCTCAGACTGTTAGTTCAG | ATACTGTTGTCCGTTACATCAC |
| MD15G1302700 | PR1 | GGTACACTCAACGGGGCC | TGTGACCACGACTTCGCC |
| MD06G1188700 | ICS1 | CAGAGGAGGCACGGCTTTTA | ATAGATCATCGCGCCGAGAC |
| MD05G1056900 | EDS5-1 | CGACGGGGTTCTTATCGGAG | AGCTTCCCTGACCAATGACG |
| MD10G1065100 | EDS5-2 | GGACTTGCTGCTCCCGTATT | GAGAGGTTCACCCCACACTG |
| MD16G1142800 | PBS3 | GGTGTTTCTGGTCAAGATTGCG | TTTGTCCGTGTCGATGCTCA |
| MD17G1133200 | NPR3g | CCTCGGCCAGTTCTCAAAGT | TACACCGATTGACACCCACG |
| MD14G1089200 | TGA2c-1 | GGTCATCGGGGAATGTTGCAAA | CGCACGAGCTGATTGTCGAGTA |
| MD14G1089200 | TGA2c-2 | AGCTTCTGATTCCAGTGA | TGTTGTAAAGTTTGTTGTAC |
| MD09G1121600 | WRKY1 | ACCACTCCATCTACTACACT | GTTCCACTTGCTTCTTCAC |
| MD17G1056000 | EPS1 | CGATCAGAAGCAATATCTCAAG | GCAAGGCAATATGTCATAGAAG |

Supplemental table S2 Gene IDs and primers of coding sequences in this study

| Gene ID | Gene name | F | R |
| --- | --- | --- | --- |
| MD00G1143500 | WRKY40 | ATGGACTCAACGTGGGTGAACA | TCACTTTTCACCATTTCGGGCC |
| MD17G1133200 | NPR3g | ATGGATAACGTCAATGAACTGTCATC | TCAATTATAAAGAGTCTGAACAAGCTGA |
| MD02G1082100 | NPR1 | ATGGGTGACGATCACTTCGTTA | TTATTTCTTGATGGTGATCGTACC |
| MD14G1089200 | TGA2c | ATGGGCAGTAGAAGAGTGAAAG | TCACTCCCTAGGCCTGGCAAGC |
| MD09G1121600 | WRKY1 | ATGATTCCTTTAGGGGAAGATGG | TTAACTGTCAACAGGTTCTGCTTTC |
| MD17G1056000 | EPS1 | ATGGCATTGAAATTCATTCAAG | TCAAAGGGTTTCAAGACCTTTA |

Supplemental table S3 Gene IDs and primers of promoter sequences for Y1H in this study

| Gene ID | Gene name | Length | F | R |
| --- | --- | --- | --- | --- |
| MD17G1133200 | NPR3g | 286 bp | TATGCAGAAAATGTGTTTACAT | AGATGATGCCCTGAAATTCAAT |
| MD00G1143500 | WRKY40 | 100 bp | ATTAATATCTTATCTAGCAAAGTCGGAGC | TGGGGATGGACTATGGAGGAG |
| MD17G1056000 | EPS1 | 143 bp | TAATGAATTGGTCCCAGCTTG | TCAATACAAGCCGGAACCCAC |

Supplemental table S4 Gene IDs and primers of promoter sequences for LUC in this study

| Gene ID | Gene name | Length | F | R |
| --- | --- | --- | --- | --- |
| MD17G1133200 | NPR3g | 817 bp | GATGCTCTAAGAAAGTAGTACG | CAGTTGGAGACAATGAATCCTC |
| MD00G1143500 | WRKY40 | 971 bp | GGCACTACATGTGCTATTGTTT | CTTAAGATTGTCACGTCTCATG |
| MD17G1056000 | EPS1 | 983 bp | CCGTATCTCAACCTTTCAGATC | TTCACTTGTTCATCCAAAATTT |

Supplemental table S5 Gene IDs and DNA probe for EMSA in this study

| Gene ID | Gene name | DNA probe |
| --- | --- | --- |
| MD17G1204800 | LRR | ACAATTCACTCTTGACTTCCAGATAGACTTT |
| MD17G1133200 | NPR3g | GTCTTGTTACTTCCAGTCAACCGTTTGCTCT |
| MD00G1143500 | WRKY40 | GTCGGAGCCTTGACCTTGACCATCAGGAAT |
| MD17G1056000 | EPS1 | AAGTCTGCCATTTGACTTTTTTTATTTGGTC |
